# Inter-Slice Representation Outweighs Bounding-Box Supervision Extent in Lightweight 2.5D Pulmonary Nodule Detection: A Whole-Volume Benchmark on LUNA16

**DOI:** 10.3390/diagnostics16183038

**Published:** 2026-09-19

**Authors:** Lien-Feng Chou, Bing-Ru Peng, Shou-Wei Chien, Yu-Ming Huang

**Affiliations:** 1 Department of Medical Imaging and Radiological Sciences, Central Taiwan University of Science and Technology, Taichung 406, Taiwan; peterbee628@gmail.com; 2 Department of Radiology, Nantou Hospital, Ministry of Health and Welfare, Nantou City 540, Taiwan; 3 Department of Information Technology, Nantou Hospital, Ministry of Health and Welfare, Nantou City 540, Taiwan; sowejan@nant.mohw.gov.tw; 4 Information Management Department, National Yunlin University of Science and Technology, Yunlin 64002, Taiwan; m11223018@yuntech.edu.tw

**Keywords:** pulmonary nodule detection, lightweight object detection, 2.5D input representation, bounding-box supervision, LUNA16, free-response operating characteristic, evaluation protocol

## Abstract

**Background/Objectives:** Lightweight detectors are attractive for high-throughput low-dose CT lung-cancer screening, yet the training-time choices governing their accuracy are usually fixed without justification, as is the protocol used to evaluate them. We benchmarked the inter-slice input representation and the extent of bounding-box supervision for 2.5D pulmonary nodule detection under the official LUNA16 protocol. **Methods:** Using one YOLO11n backbone we compared a 2D central-slice baseline, thin-slab maximum-intensity projection, and adjacent-slice 2.5D input under loose and core-focused (60% of diameter) supervision. Evaluation followed the official subset0–subset9 ten-fold protocol over all 888 scans and 1186 nodules, with whole-volume inference across 227,225 axial slices, annotations_excluded.csv applied, and paired scan-level bootstrap intervals. Supervision ratio, minimum box size, negative mining, three seeds and a YOLO26n backbone were ablated. **Results:** The representation dominated. Adjacent-slice input reached a competition performance metric (CPM) of 0.7795 (95% CI 0.7517–0.8005) against 0.6498 for the 2D baseline (+0.1297; 10 of 10 folds; d_z = 2.96), while thin-slab projection (0.5965) was worse than a plain 2D slice. Core-focused supervision gave no benefit (−0.0209) and no ratio improved on full-diameter supervision. Re-scoring the same checkpoints over only the slices holding an annotated nodule centre reversed the supervision result (+0.0027) and shrank the representation effect fourfold (+0.0348). **Conclusions:** Inter-slice representation, not supervision extent, is the dominant design factor, and the slice set searched at inference decides whether either factor is measurable.

## 1. Introduction

Lung cancer remains a leading cause of cancer-related mortality worldwide, and early detection is central to improving clinical outcomes [1]. Low-dose computed tomography (LDCT) screening has been shown to reduce lung-cancer mortality in high-risk populations, establishing CT-based screening as an important strategy for early lung-cancer detection [2]. However, CT screening generates large volumetric image sets and a substantial number of candidate findings, making pulmonary nodule detection a time-consuming task for radiologists and motivating the continued development of computer-aided detection (CAD) systems [3]. Because a screening CT yields hundreds of axial slices per study, lightweight detectors that remain computationally efficient at screening scale are of particular interest.

Deep learning has substantially advanced pulmonary nodule detection in CT. Three-dimensional convolutional neural networks (CNNs) can directly model volumetric context and have achieved strong detection performance in pulmonary nodule CAD tasks [4,5,6]; however, they typically incur higher GPU memory consumption and computational cost than their 2D counterparts, which can limit practicality in resource-constrained or rapid-screening environments. Conversely, 2D object detectors offer computational efficiency and ease of deployment, yet a single axial slice may provide insufficient through-plane information for nodules that span multiple CT slices.

Recent LUNA16-based detection studies that employ 3D CNNs, false-positive reduction networks, or multi-stage CAD pipelines have reported FROC-based detection performance in the approximate CPM range of 0.84–0.89 under the official subset0–subset9 protocol [5,6]. Representative examples include Ding et al. [5], who combined a 2D Faster R-CNN candidate generator with a 3D deep CNN false-positive reduction stage and reported a CPM of approximately 0.89, and Zhu et al. [6], who proposed DeepLung—a 3D dual-path Faster R-CNN—and reported a CPM of approximately 0.84. Other 3D-CNN-based detection pipelines on LUNA16 have likewise explored multi-stage candidate generation paired with separate false-positive reduction networks [4]. These systems typically rely on full 3D convolution and on substantially larger model capacity than the lightweight YOLO11n backbone (approximately 2.6 million parameters) evaluated in the present work. The trade-off between detection performance and model capacity therefore motivates a closer examination of which design choices in lightweight 2D/2.5D detection yield meaningful performance differences [7]. More recent work evaluated under the same official protocol has continued to raise reported performance; for example, a multi-scale 3D network with a global channel–spatial attention mechanism reports a CPM of approximately 0.93 [8].

To balance volumetric context and computational efficiency, 2.5D approaches have been widely explored in medical image analysis. Prior work has incorporated limited 3D information through multi-view sampling, random view aggregation, or multi-slice convolutional networks [9,10]. Multi-view CNN approaches have also been applied to nodule follow-up prediction tasks [11], further illustrating the utility of 2.5D representations in pulmonary CT analysis beyond initial detection. These strategies suggest that partial volumetric context can improve candidate characterisation without the full computational burden of 3D convolution. However, in lightweight object-detection frameworks, it remains unclear which 2.5D design choices are most important for pulmonary nodule detection performance.

Two practical design factors are particularly relevant. The first is the slice representation used to encode through-plane information. Thin-slab maximum-intensity projection (MIP) compresses information from adjacent slices into a single projected image, whereas direct adjacent-slice stacking preserves the ordered slice channels as separate inputs.

Although projection-based representations may visually enhance high-attenuation structures, their effect as learning inputs for a lightweight detector should not be assumed. MIP may alter through-plane intensity relationships by retaining only the maximum intensity across slices, while adjacent-slice stacking preserves the original inter-slice ordering. Whether this difference has a material effect on detection performance is an empirical question—and, as we show, one whose answer depends critically on whether the detector is evaluated over whole volumes or only over slices selected by the reference standard.

The second factor is the spatial extent of bounding-box supervision. In many object-detection pipelines, bounding boxes are generated from nodule-diameter annotations and are treated mainly as a label-conversion step. However, the box size determines which spatial locations are assigned as positive training samples during label assignment, and it also defines the regression target for bounding-box prediction [12]. A full-diameter box therefore propagates training signals from both the nodule core and its boundary region, whereas a smaller core-focused box restricts these signals to the central portion of the lesion. This distinction is important because perinodular and boundary features can carry clinically relevant information for nodule characterisation [13,14]. Nevertheless, the role of such peripheral context may differ between malignancy characterisation and candidate detection, particularly when the detector backbone is lightweight and capacity-constrained.

Together, these considerations motivate a focused, controlled benchmark. In lightweight 2.5D pulmonary nodule detection, two training-time design choices are routinely fixed without systematic justification: the inter-slice input representation and the spatial extent of the bounding box used for supervision. A third choice is fixed even more silently—the protocol under which candidate detections are generated and scored. Using the LUNA16 dataset and a single unified YOLO11n detection backbone, we benchmark the first two factors under the official LUNA16 evaluation protocol, and we show that the third factor is not neutral: with the trained models held fixed, restricting inference to the axial slices that contain an annotated nodule centre reverses the supervision conclusion outright and attenuates the representation effect to about a quarter of its size.

The main contributions of this study are as follows:

1. We present a controlled benchmark of two training-time design factors—inter-slice input representation and bounding-box supervision extent—executed under the official LUNA16 subset0–subset9 ten-fold protocol with whole-volume inference, the official irrelevant-finding exclusion list, and validation partitions disjoint from the test partitions, using a single unified backbone so that observed differences are attributable to the factors of interest.
2. We show that the inter-slice representation is the dominant factor: adjacent-slice 2.5D stacking exceeds a 2D central-slice baseline by +0.1297 CPM (95% CI +0.1061 to +0.1524) over the full cohort, and the advantage is present in every one of the ten official folds (mean fold-wise difference +0.1234, Cohen’s d_z = 2.96), whereas thin-slab MIP is inferior to a plain 2D slice.
3. We show that reducing the bounding-box supervision extent confers no benefit, and that a six-point ratio sweep combined with a minimum-box-size sweep identifies the operative quantity as the fraction of training boxes whose side is set by the minimum box constraint rather than by the annotated diameter—a quantity we formalise and which we recommend that studies sweeping a supervision ratio report.
4. We isolate the evaluation protocol as an experimental factor in its own right. Scoring the same checkpoints over ground-truth-selected slices instead of whole volumes reverses the supervision conclusion, attenuates the representation effect fourfold, and inflates every absolute CPM by 0.17 to 0.31; we also quantify the effect of the official irrelevant-finding exclusion list on CPM (+0.068 to +0.117 across configurations). Complete code, weights, candidate lists and a verified re-implementation of the official evaluation script are released publicly.

This work is a benchmarking study conducted under the official LUNA16 evaluation protocol rather than a submission to the LUNA16 challenge leaderboard. It does not address external datasets, detector architectures beyond the two lightweight YOLO backbones examined here, or clinical deployment readiness.

Interest in lightweight YOLO-family detectors for pulmonary nodule detection has continued through 2025 and 2026. Recent work has augmented YOLOv11 with wavelet convolution, attention modules and hierarchical multi-scale mixers, reporting improvements in mean average precision (mAP) on LUNA16-derived slice datasets [7]; attention-based and transformer-based multi-scale detectors have likewise reported gains on the same cohort [15,16]. These studies are informative about architectural design, but they are not directly comparable either to one another or to challenge-style results, because they differ in the unit of evaluation. Several evaluate on randomly partitioned two-dimensional slice sets rather than on the official scan-level subset partition, and report mAP rather than the free-response operating characteristic (FROC)-based competition performance metric (CPM), so their numbers describe slice-level box agreement rather than per-scan candidate detection. The present study is deliberately positioned differently: it fixes the backbone, adopts the official scan-level protocol without modification, and varies only the training-time factors under study.

Reported LUNA16 numbers are only comparable when they arise from the same protocol—the official subset partition, whole-volume candidate generation, and the official irrelevant-finding exclusion list. A comparison that does not state these attributes invites conclusions the numbers do not support, so we defer it to Section 3.13, which lists each published entry together with the protocol that produced it and a flag stating whether it is commensurable with the values reported here.

## 2. Materials and Methods

### 2.1. Dataset and Evaluation Protocol

This study used the LUNA16 dataset, a public pulmonary nodule detection dataset derived from the LIDC-IDRI cohort [17,18] and containing 888 thoracic CT scans with expert nodule annotations [3]. The dataset was used as a retrospective, de-identified imaging cohort. All evaluation follows the official LUNA16 protocol.

Cross-validation partition. Evaluation used the official subset0–subset9 partition. For fold *k* (k = 0,…,9) the test partition is subset k; the validation partition, used only for checkpoint selection and early stopping, is subset (k + 1) mod 10; and the remaining eight subsets form the training partition. Subset sizes are 89 scans for subsets 0–7 and 88 scans for subsets 8 and 9, totalling 888 scans. Carving the validation partition out of the training subsets ensures that no scan influences the model that is later evaluated on it, either through gradient updates or through model selection. An audit confirming zero overlap between the training, validation and test partitions of every fold is released with the code. Out-of-fold predictions were concatenated, so each of the 888 scans is scored exactly once.

Reference standard and irrelevant findings. Detection results were evaluated against the 1186 nodules of annotations.csv. Candidates falling inside an entry of annotations_excluded.csv—the official list of 35,192 irrelevant findings, which covers all 888 scans at a mean of 39.6 findings per scan—were ignored rather than counted as false positives, following the official rule. Both files were obtained from the official LUNA16 evaluation archive distributed at Zenodo [19] (doi:10.5281/zenodo.3723295); the annotations.csv obtained there is byte-identical to the copy used in our earlier analysis.

Of the 35,192 irrelevant findings, 30,513 carry a placeholder diameter of −1 mm. The evaluation script as currently distributed replaces a negative diameter with 10 mm, giving a 5 mm ignore sphere, and we adopt that convention throughout because it is what any reader running the script today obtains. The choice is not immaterial: evaluating the placeholders at their absolute value instead lowers CPM by 0.034 to 0.050 across the four configurations, more than the difference between the two supervision settings. Both conventions are implemented and both are reported in Section 3.3.

Evaluation metric. The primary endpoint was the competition performance metric (CPM), the mean sensitivity at seven false-positive-per-scan operating points, following the LUNA16 free-response receiver operating characteristic framework [3,20]. Let S be the 888-scan cohort, G the 1186 reference nodules and E the irrelevant findings. A candidate c hits an annotation a when its centre falls inside the annotated ball,

hit(c, a) ⟺ ‖x_c − x_a‖^2^ < (d_a/2)^2^, with d_a ← 10 mm if d_a < 0 (1)

Each scan’s candidates are then partitioned, in this order, into one true positive per hit nodule, further hits on an already-claimed nodule, hits on an irrelevant finding, and the remainder,

C = C_TP ⊔ C_dup ⊔ C_irr ⊔ C_FP(2)

where C_TP takes the highest-scoring hit of each nodule, q_g = max{p_c: hit(c, g)}. C_dup and C_irr are discarded rather than scored—this is the role of annotations_excluded.csv, and omitting it inflates the false-positive count by |C_irr|. Sweeping a threshold t over the retained scores gives

Sens(t) = |{g ∈ G: q_g ≥ t}|/|G|, FP/scan(t) = |{c ∈ C_FP: p_c ≥ t}|/|S|(3)

and eliminating t yields the FROC curve F. CPM is that curve sampled at seven fixed operating points,

CPM = (1/7) Σ_{η ∈ H} F(η), H = {1/8, 1/4, 1/2, 1, 2, 4, 8}(4)

with F linearly interpolated at each η. Equation (3) divides by |S| = 888 whether or not a scan produced candidates, so whole-volume inference over all 888 scans—including the 287 with no reference nodule—is a precondition for Equation (4) to mean what it says.

Evaluator verification. We re-implemented the official evaluation script noduleCADEvaluationLUNA16.py in Python 3 and verified it against CADAnalysis.txt, the reference output distributed with the same archive. That reference predates the negative-diameter fallback described above, so reproducing it requires evaluating the placeholder diameters at their absolute value; under that convention our implementation reproduces every published counter exactly on the supplied sample submission: 1120 true positives, 548,420 false positives, 66 false negatives, 551,065 total candidates, 1186 nodules, 1294 candidates ignored on excluded findings and 231 ignored double detections. The agreement establishes that the matching, ignoring and FROC construction are faithful; the results reported in this paper then apply the 10 mm fallback of the current script, under which 6555 rather than 1294 candidates are ignored on that submission. The verification runs as an automated test in the released code and requires no imaging data.

### 2.2. CT Preprocessing and Input Construction

All CT volumes were preprocessed identically. Voxel intensities were clipped to a Hounsfield unit window of −1000 to 400 HU and linearly normalised to an 8-bit range. Training and inference were performed at the native in-plane resolution of 512 × 512 pixels. The earlier version of this analysis resampled slices to 640 × 640, the default input size of the detector; operating at native resolution removes that interpolation and makes one pixel correspond to one voxel, so the minimum box constraint of Section 2.3 has an exact and scan-independent meaning.

**Figure 1:**
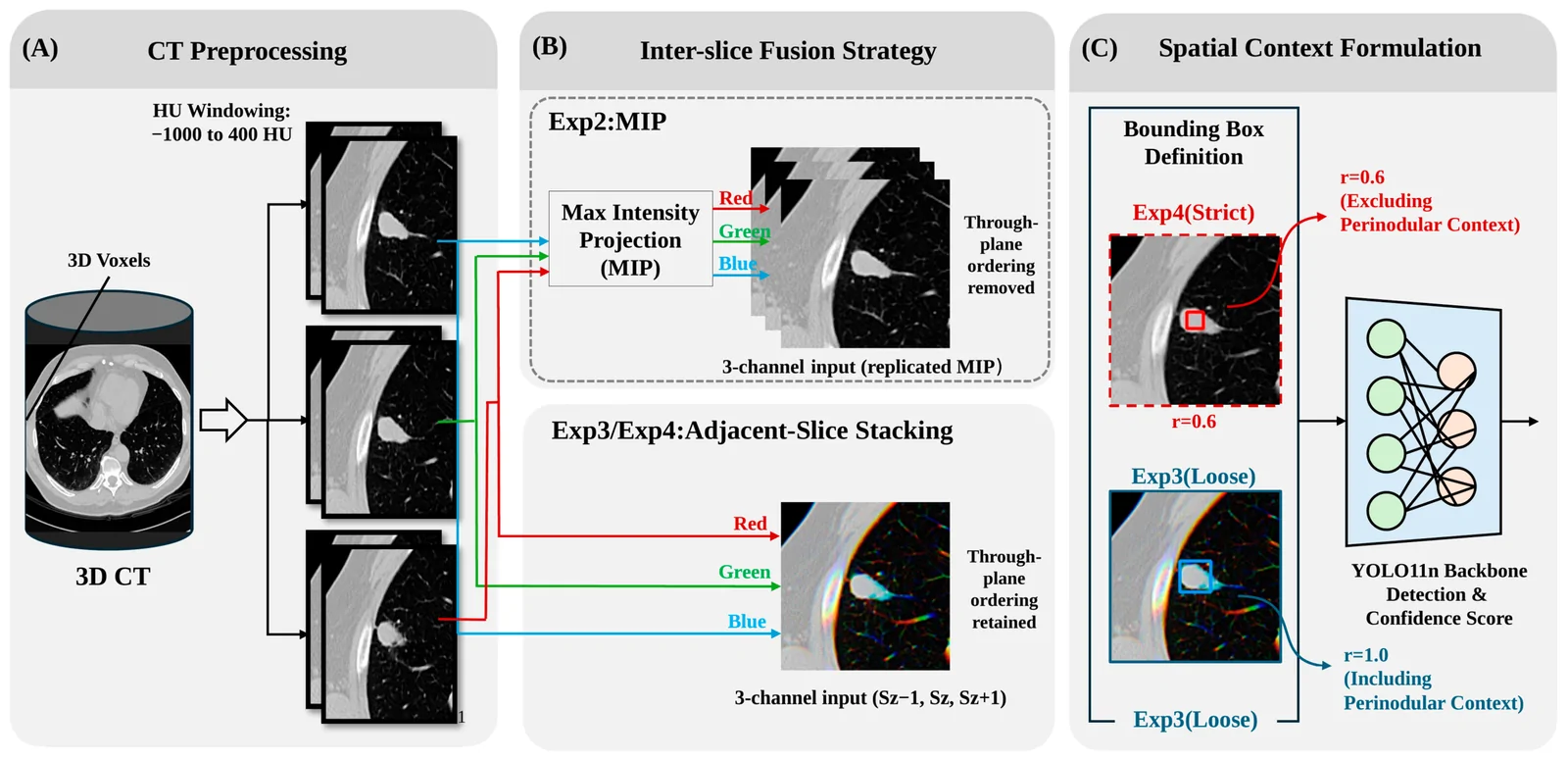
Preprocessing and study design. (**A**) Volumes are windowed to [−1000, 400] HU and rescaled to 8 bits at the native 512 × 512 in-plane resolution. (**B**) Inter-slice representations: the 2D baseline replicates the central slice across the three input channels; the thin-slab maximum-intensity projection compresses the slab {S_z − 1, S_z, S_z + 1} into one plane, which is then replicated; adjacent-slice stacking assigns the three slices to the three channels, preserving the through-plane ordering. (**C**) Supervision geometry. A nodule is modelled as a sphere, so the box side on each intersected slice follows the chord diameter of Equation (8), scaled by the supervision-extent ratio r and floored at w_min (Equation (9)). Two regimes follow from Equation (10). Below the threshold d × (r, w_min) the strict box is set by the floor rather than by the ratio; below the smaller threshold d × (1, w_min) = w_min s_xy the loose box is clamped as well, and only there are the two labels identical. Between the two thresholds the nominal ratio overstates the contrast actually applied, which is why the clamped fraction of Equation (11) is reported alongside r throughout. The arrows represent channel assignment and the red/blue boxes represent Exp4 strict r = 0.6/Exp3 loose r = 1.0.

The preprocessing pipeline, the three inter-slice representations and the supervision geometry of Section 2.3 are summarised in Figure 1.

Three inter-slice representations were compared, all defined on the same slice index so that they differ only in how through-plane information is encoded (Equation (7) below). Exp1 uses the central axial slice replicated across the three input channels. Exp2 uses a maximum-intensity projection (MIP) over the three-slice slab {S_{z − 1}, S_z, S_{z + 1}}, likewise replicated. Exp3 and Exp4 assign the three adjacent slices directly to the three channels, preserving the through-plane ordering. Exp3 and Exp4 differ only in the supervision extent of Section 2.3.

### 2.3. Mathematical Framework and Bounding-Box Supervision

The complete derivation of the framework below—Equations (1)–(15), with all intermediate steps, the voxel-to-world convention shared by label generation and scoring, the clamping analysis, and the definition of the paired scan-level bootstrap—is provided in the Supplementary Materials (Supplementary File S1).

Geometry. A scan is a scalar field on a voxel lattice with spacing **s** = (s_x, s_y, s_z), origin **o** and direction cosine matrix D. Voxel indices and world coordinates are related by

φ(**v**) = **o** + D(**v** ⊙ **s**), φ^−1^(**x**) = D^−1^(**x** − **o**) ⊘ **s** (5)

Both label generation and candidate scoring use φ and φ^−1^, so a training target and the evaluation target it corresponds to are the same physical point.

Intensity normalisation. With the window [h_min, h_max] = [−1000, 400] HU,


(6) $$\tilde{S}(v)=\lfloor 255\;\cdot \;(\mathrm{clip}(S(v),h\text{\_}\min,h\text{\_}\max)-h\text{\_}\min)\text{/}(h\text{\_}\max-h\text{\_}\min)\rfloor$$

Representation operators. With $\tilde{S}$_z the normalised axial slice z,

(7) $$\Psi \text{\_}2D(z)=[\tilde{S}\text{\_}z,\tilde{S}\text{\_}z,\tilde{S}\text{\_}z],\Psi \text{\_}\mathrm{MIP}(z)=[M\text{\_}z,M\text{\_}z,M\text{\_}z]\mathrm{with}M\text{\_}z=\max\text{\_}\{|k|\leq 1\}\tilde{S}\text{\_}\{z+k\},\Psi \text{\_}2.5D(z)=[\tilde{S}\text{\_}\{z-1\},\tilde{S}\text{\_}z,\tilde{S}\text{\_}\{z+1\}]$$

Ψ_2D and Ψ_MIP both produce channel-constant images, so whatever through-plane information they carry must already have been compressed into a single plane: the 2D operator discards the adjacent slices entirely, and the projection retains only the voxel-wise maximum, losing both the ordering of the slab and every value below that maximum. Ψ_2.5D is the only one of the three whose output distinguishes the three slices, and it is therefore the only one from which a detector can learn a through-plane relation.

Supervision extent. A nodule of annotated diameter d is modelled as a sphere. The axial plane at through-plane offset δ_z millimetres from the centre intersects it in a disc of chord diameter

d_eff(z) = 2 √((d/2)^2^ − δ_z^2^), defined for |δ_z| ≤ d/2 (8)

and the training box on that slice is a square of side

w_label(z; r, w_min) = max( r · d_eff(z)/s_xy, w_min)(9)

in native pixels, where s_xy is the in-plane voxel spacing, which is isotropic in every scan of this cohort (median 0.703 mm, range 0.461–0.977 mm), r is the supervision-extent ratio (r = 1 loose, r < 1 core-focused) and w_min the minimum admissible side. Where the floor does not bind, the supervised axial area scales as r^2^, so r = 0.6 covers 36% of the loose area; where it binds, the box side is w_min irrespective of r, and the ratio has no effect on that box. It does not follow that the strict and loose labels agree there: they agree only where both are clamped, a distinction developed next.

The clamping regime. Equation (9) is piecewise. The floor binds exactly when

r · d_eff(z)/s_xy < w_min ⟺ d_eff(z) < w_min · s_xy/r =: d × (r, w_min)(10)

Because d× is proportional to w_min/r, the two parameters are not independent factors. Lowering r raises d×, enlarging the subpopulation whose label is set by the floor rather than by the diameter, and the two supervision settings therefore coincide over part of that subpopulation: a strict label and a loose label are equal precisely when both are clamped, that is for d_eff < d × (1, w_min) = w_min · s_xy. Between that threshold and d × (r, w_min) only the strict label is clamped, so the nominal ratio r overstates the contrast actually applied. We therefore define the clamped fraction

ρ(r, w_min) = |{b ∈ B: d_eff(z_b) < d × (r, w_min)}|/|B|(11)

over the set B of training boxes, where z_b is the slice on which box b lies, and report it for every cell of the design grid (Section 3.5). With w_min = 6 px, ρ rises from 0.186 at r = 1.0 to 0.520 at r = 0.6 and 0.723 at r = 0.4; at fixed r = 0.6 it moves from 0.249 at w_min = 4 px to 0.671 at w_min = 8 px. Reporting ρ is what makes a supervision ratio sweep interpretable, because a change in performance across r is otherwise confounded with a change in the mixture of diameter-driven and floor-driven boxes.

The minimum box side is not a modelling choice but a numerical one: anchor-free detectors assign targets dynamically from box geometry [12], and a box of a few pixels destabilises that assignment. Equation (10) is what makes the cost of imposing it visible.

Labelled slices. Boxes were generated on every axial slice the sphere intersects, not only on the centre slice. Whole-volume inference requires supervision on every slice at which the lesion is visible, and Equation (8) defines the box there without introducing a further free parameter.

### 2.4. Model Architecture and Training

YOLO11n [21] was used as the unified lightweight backbone for all primary configurations, with YOLO26n evaluated as a second backbone (Section 3.9). All configurations shared one training schedule, pinned explicitly rather than left to framework defaults: 35 epochs, batch size 64, input size 512 × 512, AdamW [22] with initial learning rate 0.001, final learning-rate factor 0.01, weight decay 0.0005, three warm-up epochs and early-stopping patience of 15. Augmentation comprised mosaic with probability 0.5, disabled for the final ten epochs, random translation up to 10%, scaling within 30%, horizontal and vertical flips at probability 0.5, and brightness jitter of 0.2. No rotation, shear, perspective transformation, mix-up or random erasing was applied, and hue and saturation jitter were set to zero because the three input channels encode through-plane position rather than colour, so perturbing them would mix information between slices. Models were initialised from the publicly available COCO-pretrained weights with a re-initialised single-class detection head. The training seed was set explicitly for every run.

The training pool comprised 21,377 axial slices per representation, of which 7169 were nodule-bearing and the remainder background, drawn from all 888 scans including the 287 that contain no reference nodule. Background slices were eligible only if they contained lung parenchyma and lay outside the through-plane extent of every annotation plus a two-slice guard, so a sampled background slice cannot contain an unlabelled reference nodule.

The four primary configurations and the negative-mining ablation were trained on all ten official folds. The parameter sweeps, the multi-seed repetition and the second backbone are sensitivity analyses rather than primary endpoints, and were trained on the first five folds (the first three for the count-matched negative arm) to keep the 123-run matrix within the available compute. Every table states the number of scans it covers, and all configurations entering a given table are scored on the cohort common to them, so no comparison is made across different evaluation cohorts.

Experiments were run on a single workstation with an NVIDIA RTX PRO 6000 Blackwell GPU (NVIDIA Corporation, Santa Clara, CA, USA; 17.2 GB of device memory), an Intel Xeon (Sapphire Rapids) CPU and 32 GB of system memory, using Python 3.10, PyTorch 2.12.0 (CUDA 13.0) and Ultralytics 8.4.117 [23]. At 512 × 512 input, YOLO11n has 2,624,080 parameters and 4.26 GFLOPs, a mean single-slice latency of 11.91 ± 1.08 ms and a batched throughput of 268.6 slices/s, corresponding to 0.95 s for a 256-slice scan. YOLO26n has 2,572,280 parameters and 3.97 GFLOPs, a latency of 12.36 ± 1.15 ms and a throughput of 382.3 slices/s (0.67 s per scan); its higher single-slice latency alongside higher batched throughput is consistent with its end-to-end head, which removes non-maximum suppression from the per-image path but does not shorten the fixed per-call overhead. Latencies were measured over 200 consecutive axial slices of a real LUNA16 scan held in memory, at batch size 1, timing Ultralytics preprocessing, the forward pass and post-processing together, with CUDA synchronisation around every call; throughput was measured at batch size 64. The full profile, including the hardware string and library versions, is released as results/model_profile.json.

### 2.5. Whole-Volume Inference and Candidate Aggregation

Whole-volume inference. For each evaluation scan the trained detector was applied to every axial slice of the volume—227,225 slices across the 888-scan cohort—at a permissive confidence threshold τ_0_ = 0.01, slice-level non-maximum suppression (NMS) at an intersection-over-union (IoU) threshold of 0.45, and at most 30 detections per slice. No use was made of the reference standard in selecting which slices to evaluate.

Aggregation. Slice-level detections were aggregated into 3D candidates greedily in descending confidence. Order the detections of a scan so that p_1_ ≥ p_2_ ≥ …, and let each candidate c be represented by its most confident member (z_c, b_c, p_c). Detection j joins candidate c when

|z_j − z_c| ≤ Δ_z and IoU(b_j, b_c) ≥ τ_IoU(12)

and otherwise seeds a new candidate. Because detections arrive in non-increasing confidence the representative never changes after a candidate is created, so the candidate score is

p_c = max_{j ∈ c} p_j(13)

The candidate centre is mapped back to world coordinates with the map φ of Equation (5),

x_c = φ((u_c·N_x − ½, v_c·N_y − ½, z_c))(14)

which is the exact inverse of the encoding used to write the training labels, so no half-voxel bias is introduced between supervision and scoring. The constants were τ_0_ = 0.01, Δ_z = 3 slices and τ_IoU = 0.1. At most, 150 candidates were retained per scan, an order of magnitude above what the 8 FP/scan operating point can consume. These parameters were identical for every configuration, so none of them can favour one configuration over another.

### 2.6. Statistical Analysis

Uncertainty in CPM was estimated by scan-level bootstrap resampling with B = 1000 iterations and percentile intervals. Let S^(b)^ be the b-th resample, drawn from S with replacement and of the same size, and CPM_m^(b)^ configuration m’s CPM recomputed on it. All configurations were scored on the same resamples, so the replicates of a difference

Δ^(b)^ = CPM_m^(b)^ − CPM_{m′}^(b)^ (15)

are paired by construction; the reported interval for a contrast is the percentile interval of Δ^(b)^ and the reported *p*-value is 2·min(Pr[Δ^(b)^ ≤ 0], Pr[Δ^(b)^ ≥ 0]). Non-overlap of two marginal intervals, which is a weaker statement, is not used as evidence anywhere in this paper.

The FROC curve is obtained from sklearn.metrics.roc_curve with its default drop_intermediate = True, as in the official script. On the candidate volumes reported here, recomputing the curve with all threshold points retained changes no CPM value in the sixth decimal place.

Partition sensitivity was assessed separately by computing CPM within each official fold’s own test subset and comparing configurations with a paired Wilcoxon signed-rank test, a paired t-test and the paired effect size d_z = mean(δ)/sd(δ); *p*-values across the family of primary contrasts were adjusted by the Holm–Bonferroni procedure [24]. Training stochasticity was assessed by repeating the two adjacent-slice configurations with three seeds and reporting the mean and standard deviation of CPM. These three sources of variability—evaluation-sample noise, partition dependence and optimisation noise—answer different questions and are reported separately rather than combined.

### 2.7. Relationship to the Previously Reported Analysis

An earlier version of this analysis reported higher absolute CPM values and the opposite ordering for two of the factors studied here. Adopting the official evaluation protocol changed those conclusions, and while implementing that change we audited the earlier pipeline in full. Five discrepancies were identified, all of which are corrected in the present analysis and all of which we state explicitly so that readers can reconcile the two sets of numbers.

First, and most consequentially, the earlier inference stage was applied only to the pre-generated training-pool slices—the slices containing annotated nodule centres, together with a small number of sampled background slices—rather than to whole volumes. Candidate lists therefore covered a mean of 2.6 distinct slices per scan, and the 287 nodule-free scans produced no candidates at all while remaining in the false-positive-per-scan denominator. Because the evaluated slices were selected using the reference standard, the resulting statistic was not a whole-volume FROC measure. Section 2.5 now describes true whole-volume inference.

Second, the fold configuration used the test partition as the validation partition, so checkpoint selection and early stopping were performed on the evaluation data. The official protocol of Section 2.1 uses a validation subset disjoint from both training and test partitions.

Third, two folds of one configuration terminated before completing the training schedule yet contributed to the pooled result for that configuration. All configurations reported here completed an identical schedule, and per-run epoch counts are released with the code.

Fourth, training labels were keyed on the pair (series, slice) and rewritten within the per-annotation loop, so a second nodule on the same slice replaced the first; 1186 annotations produced 1176 label entries. Boxes are now accumulated per slice before writing; the rebuilt pools contain 7466 boxes.

Fifth, the two directions of the voxel-to-world map did not agree. Labels were written with a normalisation by the image side, while candidate centres were decoded with a normalisation by the side minus one, and the direction cosine matrix was applied when scoring but not when labelling. The resulting offset between the supervision target and the scoring target is sub-millimetre, but it is not negligible against the matching tolerance: the smallest annotated nodule in the cohort has a diameter of 3.25 mm, so the tightest matching radius is 1.6 mm. A single affine map is now used in both directions (Equation (5)).

In addition, the augmentation policy, random seed, input resolution and profiling sample size stated in the earlier Methods did not correspond in every detail to the runs that produced the earlier results. Every hyper-parameter reported in Section 2.4 is now pinned in a single configuration file that is released with the code and recorded in each run’s output.

## 3. Results

### 3.1. Detection Performance Under the Official Protocol

**Figure 2:**
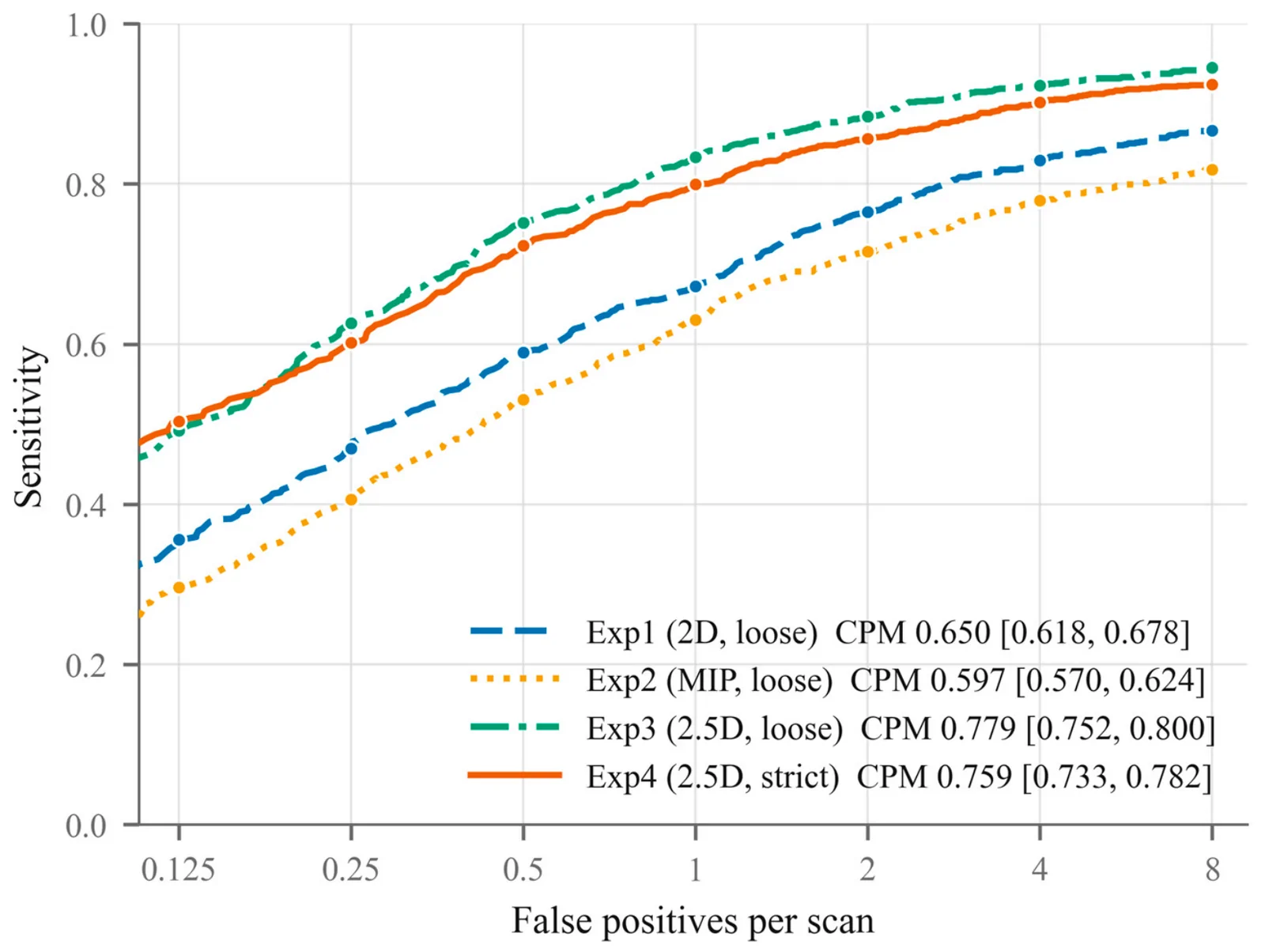
FROC curves for the four configurations under the official LUNA16 protocol, on a logarithmic false-positive-per-scan axis. Filled markers denote the seven CPM operating points; a vertical tick marks the point at which a configuration’s retained candidate pool is exhausted, and any operating point beyond it is drawn as an open marker on a dotted extension to indicate that CPM holds the final sensitivity there rather than measuring it. On these data every candidate pool extends past 8 FP/scan, so all seven markers are filled and no operating point is extrapolated. The legend gives each configuration’s CPM with its 95% scan-level bootstrap interval. Series carry distinct dash patterns as well as colours so the figure remains readable in greyscale and under common colour-vision deficiencies.

Table 1 reports CPM for the four configurations over the full 888-scan cohort, and Figure 2 shows the corresponding FROC curves. The adjacent-slice 2.5D loose configuration (Exp3) achieved the highest CPM at 0.7795 (95% CI 0.7517–0.8005), followed by the adjacent-slice 2.5D strict configuration (Exp4) at 0.7586 (0.7332–0.7821), the 2D central-slice baseline (Exp1) at 0.6498 (0.6181–0.6778) and the thin-slab MIP configuration (Exp2) at 0.5965 (0.5703–0.6239).

**Figure 3:**
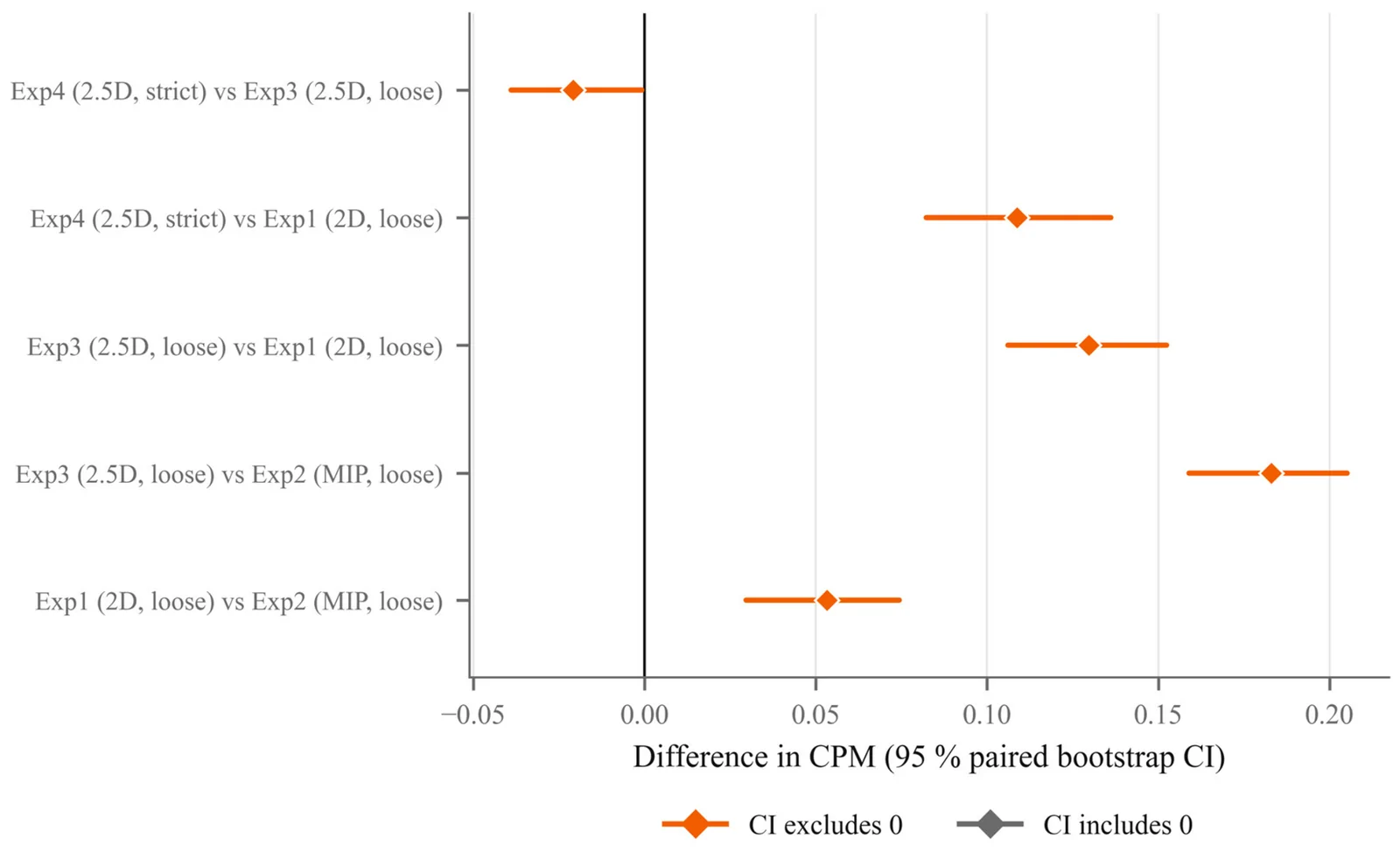
Paired bootstrap differences in CPM for the primary contrasts over all 888 scans, with 95% percentile intervals. Intervals excluding zero are highlighted. Differences are computed on shared bootstrap resamples, so each interval is a paired interval on the difference rather than a comparison of two marginal intervals.

The dominant effect is the inter-slice representation. Under matched loose supervision, adjacent-slice stacking exceeded the 2D baseline by a paired difference of +0.1297 CPM (95% CI +0.1061 to +0.1524) and exceeded thin-slab MIP by +0.1830 (+0.1590 to +0.2051). Thin-slab MIP was inferior even to a single 2D slice, by −0.0533 (95% CI of the Exp1 − Exp2 difference +0.0297 to +0.0744). All three contrasts exclude zero. Figure 3 shows all primary paired differences with their intervals.

The effect of supervision extent was roughly six times smaller and in the opposite direction to that previously reported: holding the adjacent-slice representation fixed, strict core-focused supervision was associated with a paired difference of −0.0209 CPM against loose supervision (95% CI −0.0390 to −0.0008). The interval excludes zero, but only marginally, and the fold-wise analysis of Section 3.4 does not support a consistent per-partition effect; we therefore interpret this contrast as providing no evidence that core-focused supervision improves detection, rather than as evidence that it degrades it.

### 3.2. Seven-Point FROC Analysis

Table 2 lists sensitivity at each of the seven CPM operating points. Exp3 attained the highest sensitivity at six of the seven points, and its margin over the 2D and MIP baselines was present across the entire operating range rather than confined to one threshold. At 1 false positive per scan, sensitivity was 0.8334 for Exp3, 0.7993 for Exp4, 0.6721 for Exp1 and 0.6304 for Exp2. Exp4 exceeded Exp3 only at the leftmost operating point (0.5037 versus 0.4921 at 0.125 FP/scan), and fell behind at every other point.

The retained candidate pool reached the 8 FP/scan operating point for all four configurations, so no operating point required extrapolation beyond the measured curve. Upper-bound recall over the retained pool was 0.9755 for Exp3, 0.9680 for Exp4, 0.9503 for Exp1 and 0.9030 for Exp2.

### 3.3. Effect of the Official Irrelevant-Finding Exclusion List

Table 3 quantifies the effect of applying annotations_excluded.csv. Ignoring candidates that fall on irrelevant findings removed between 6.6% and 9.4% of false positives, and raised CPM by between +0.068 and +0.117 depending on configuration: Exp1 from 0.5675 to 0.6498, Exp2 from 0.5288 to 0.5965, Exp3 from 0.6779 to 0.7795 and Exp4 from 0.6414 to 0.7586.

Table 3 also reports CPM under the pre-fallback convention for the placeholder diameters (Section 2.1). Interpreting them at their absolute value rather than as 10 mm lowers CPM by 0.0335 to 0.0497 depending on configuration, without changing the ordering.

The exclusion list also constrains what the false positives can be. Taking the candidates above the two-false-positives-per-scan operating point and partitioning them as in Equation (2), the responses that are not true positives divide into those that land inside an irrelevant finding—a structure at least one of the four readers marked and the reference standard then set aside, namely a nodule below 3 mm, a non-nodular finding, or a nodule of 3 mm or more that fewer than three readers marked—and those that land where no reader annotated anything. Between 38.2% and 47.7% of non-nodule responses fall in the first class, and of the remainder a further 10.3% to 13.5% lie within 10 mm of an irrelevant finding. The proportion is highest for the adjacent-slice configurations (47.7% for Exp3 against 40.8% for the 2D baseline), consistent with the through-plane channels making the detector more responsive to focal structures in general, including those the protocol discards. The per-configuration composition and the 200 highest-scoring counted false positives per configuration are released with the code, each with its world coordinates, its distance to the nearest annotation of either kind, and three measurements of its surroundings (distance to the lung boundary, local soft-tissue fraction, and elongation of the soft-tissue component it lies on), so that the anatomical review this analysis stops short of can be carried out on our candidates. Those measurements are reported per candidate and are not compared across configurations, because each configuration’s ranked list is drawn from a different subset of scans.

Two points follow. First, the magnitude of the correction is large relative to the differences between configurations, so an analysis that omits the exclusion list is not comparable with challenge-style results. Second, the correction is not a constant offset: it differed by 0.049 CPM between the most and least affected configuration. The ordering of the four configurations happens to be preserved here, but the size of the contrasts is not: the Exp3-minus-Exp4 difference falls from 0.0365 without the exclusion list to 0.0209 with it, a reduction of 43%. An analysis that omits the list therefore misstates effect sizes even when it recovers the correct ranking, and cannot be assumed to recover the ranking in general.

### 3.4. Fold-Wise Analysis

**Figure 4:**
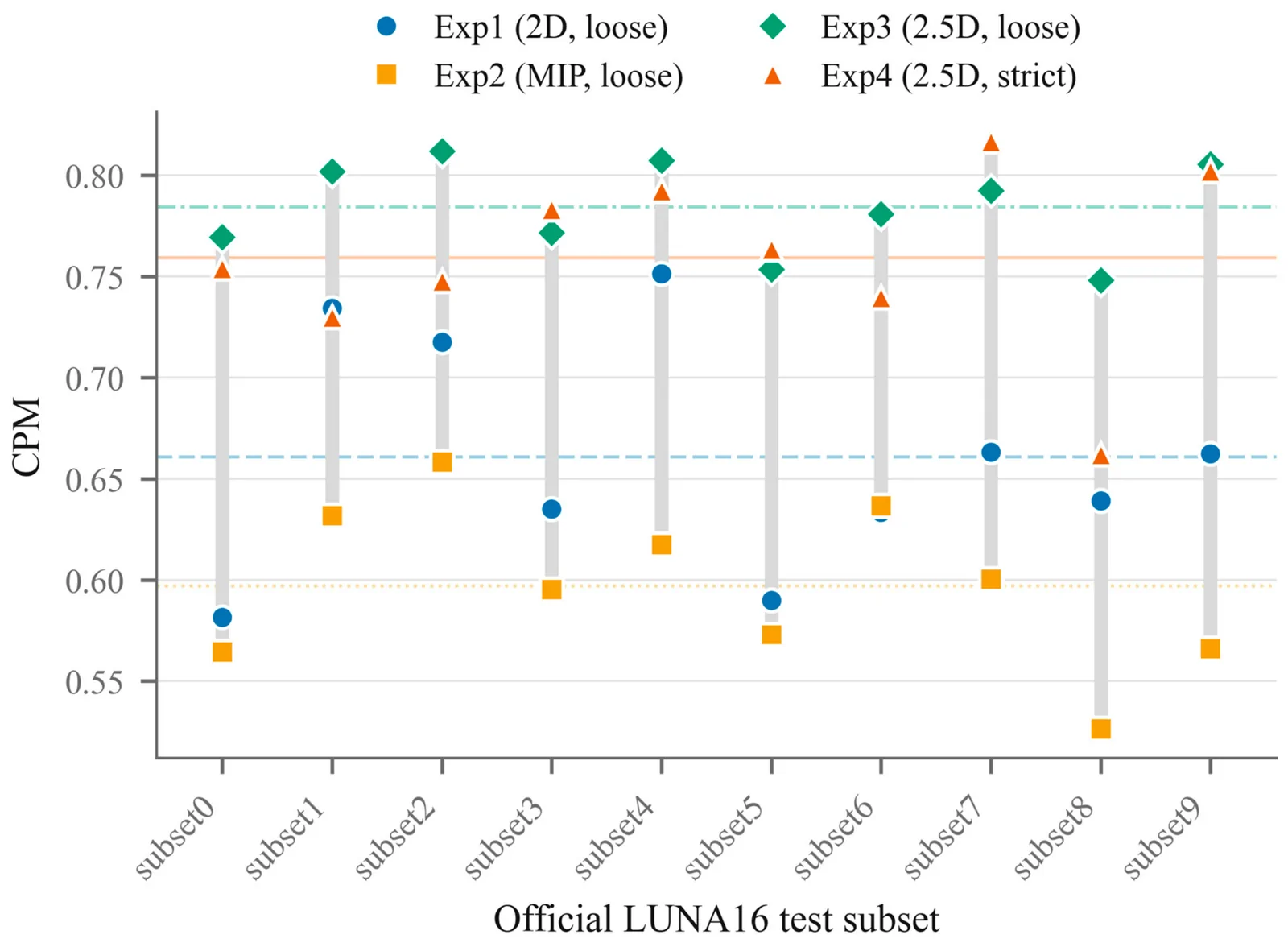
CPM computed within each official test subset, all 888 scans. Markers are drawn at the value rather than as bars, so the axis can be scaled to the data without a truncated baseline; the grey guide joins the four configurations within a fold and the faint horizontal lines mark each configuration’s mean across folds. The advantage of the adjacent-slice representation is present in all ten folds; the supervision-extent contrast is not.

Table 4 and Figure 4 report CPM computed within each official test subset. The representation effect was consistent across partitions: Exp3 exceeded Exp1 in 10 of 10 folds (mean difference +0.1234, Wilcoxon *p* = 0.0020, Holm-adjusted *p* = 0.0098, d_z = 2.96) and exceeded Exp2 in 10 of 10 folds (mean +0.1872, Holm-adjusted *p* = 0.0098, d_z = 6.40). Exp4 exceeded Exp1 in nine of 10 folds (Holm-adjusted *p* = 0.0117, d_z = 1.43).

The supervision-extent contrast behaved differently. Exp4 exceeded Exp3 in only three of 10 folds, with a mean fold-wise difference of −0.0251, a median of −0.0151 and a non-significant Wilcoxon test (*p* = 0.105, d_z = −0.65). The scan-level paired interval of Section 3.1 and the fold-wise test therefore give different answers, and they measure different things: the former quantifies sensitivity to which scans are evaluated, and the latter to which scans are used for training. We report both and base no claim on the contrast beyond the absence of evidence for a benefit.

### 3.5. Supervision-Extent Sweep and the Clamped Fraction

**Figure 5:**
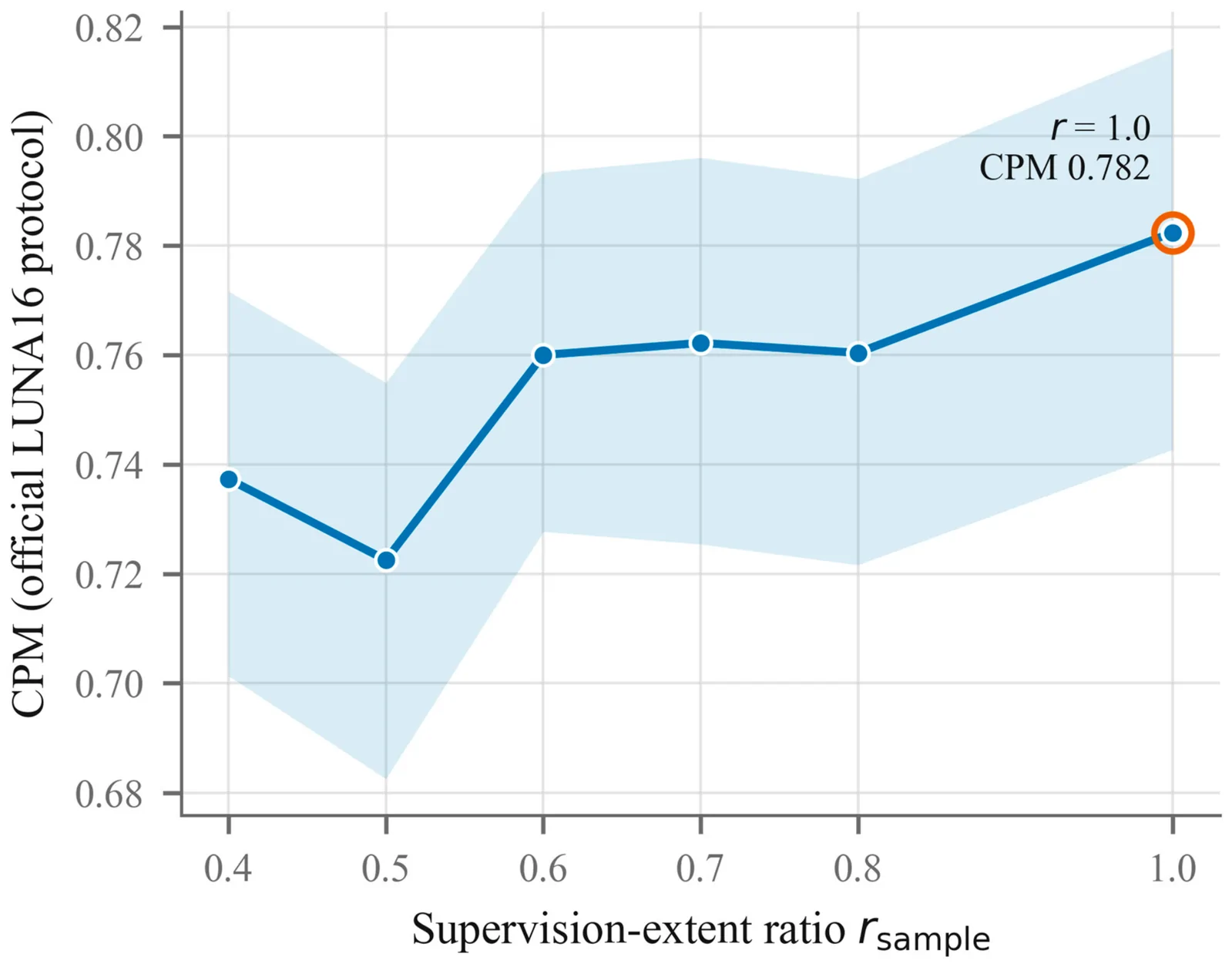
CPM against the supervision-extent ratio r, with 95% scan-level bootstrap intervals, on the 445-scan cohort common to the sweep configurations. The best point is ringed. The response is non-monotone; Table 5 relates it to the clamped fraction of Equation (11).

Table 5 tabulates the (r, w_min) design grid used throughout this section, and Table 6 and Figure 5 report the supervision ratio sweep over r ∈ {0.4, 0.5, 0.6, 0.7, 0.8, 1.0}, evaluated on the 445-scan cohort common to all sweep configurations. CPM was highest at full-diameter supervision (0.7824), formed a plateau near 0.760 for r ∈ {0.6, 0.7, 0.8}, and fell to 0.7225 at r = 0.5 and 0.7373 at r = 0.4. Relative to r = 0.6, the paired differences were −0.0375 for r = 0.5 (95% CI −0.0638 to −0.0183) and −0.0227 for r = 0.4 (−0.0448 to −0.0041), both excluding zero, whereas r = 0.7, r = 0.8 and r = 1.0 did not differ significantly from r = 0.6. No ratio improved on full-diameter supervision, and r = 0.6 was not optimal.

One consequence of evaluating the sweep on its common cohort deserves comment. On this 445-scan cohort the Exp3-versus-Exp4 contrast is +0.0224 CPM with an interval spanning zero (−0.0091 to +0.0476), whereas on the full 888-scan cohort of Section 3.1 the same contrast is −0.0209 with an interval that excludes zero. The direction is the same in both; the interval is wider on half the cohort, as expected. We draw no conclusion that depends on which side of the conventional threshold this contrast falls, and the statement we do make—that there is no evidence core-focused supervision improves detection—holds under both.

The response is not monotone, and the minimum box sweep of Section 3.6 shows why interpreting it in terms of r alone is inadequate. Table 5 reports, for every cell of the (r, w_min) grid, the clamped fraction ρ of Equation (11). Ordering the eight configurations by ρ, CPM declines from 0.7824 at ρ = 0.186 to 0.7253 at ρ = 0.671. Across the grid, CPM correlated with ρ at Pearson r = −0.880 (95% CI −0.978 to −0.463, *p* = 0.0039) and Spearman ρ_s = −0.881 (*p* = 0.0039); with the supervision ratio at r = +0.765 (95% CI +0.132 to +0.955, *p* = 0.027); with the minimum box side at r = −0.487 (95% CI −0.887 to +0.332, *p* = 0.221); and not at all with the number of false positives (r = +0.173, *p* = 0.682) or with the number of candidates per scan (r = +0.139, *p* = 0.744).

These correlations are computed over the eight cells of the design grid, so they are descriptive of that grid rather than inferential about the design space, and their intervals are correspondingly wide; the eight cells are also not independent, since ρ is a deterministic function of r and w_min given the cohort. We therefore draw only the ordinal conclusion the intervals support: of the three candidate predictors, the clamped fraction is the one whose interval excludes zero by the widest margin and the only one that orders the grid monotonically, whereas the size of the candidate pool is not predictive at all—the latter being consistent with FROC consulting only the highest-scoring candidates, so that surplus low-scoring candidates cannot affect CPM.

### 3.6. Minimum Box Size

**Figure 6:**
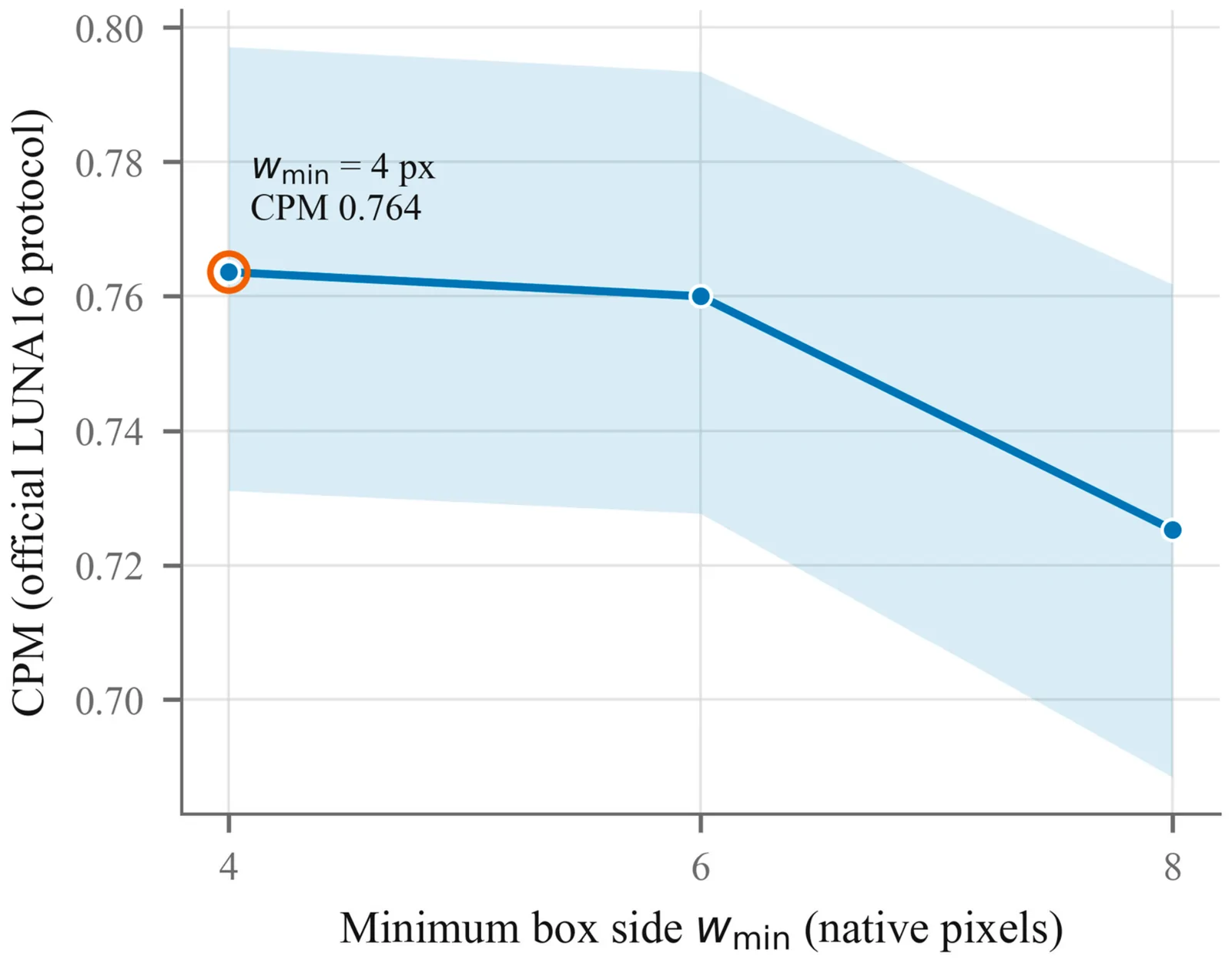
CPM against the minimum box side w_min at fixed r = 0.6, with 95% scan-level bootstrap intervals, on the same 445-scan cohort as Figure 5. Varying this constant alone moves CPM over a range comparable to that of the supervision ratio sweep.

Table 7 and Figure 6 report the minimum box sweep at fixed r = 0.6. CPM was 0.7636 at w_min = 4 px, 0.7600 at 6 px and 0.7253 at 8 px. The paired difference between 4 px and 6 px was +0.0037 (95% CI −0.0196 to +0.0271, not significant), whereas 8 px was worse than 6 px by −0.0347 (−0.0587 to −0.0104), excluding zero.

Varying only the minimum box side, at a fixed supervision ratio, moved CPM over a range comparable to that produced by the supervision ratio itself. A constant that detection pipelines impose to keep label assignment numerically stable, and that is rarely reported, therefore exerts an influence of the same order as the factor under deliberate study. The effect is not confined to sub-5 mm nodules. Equation (10) makes the strict and loose labels identical only below d × (1, w_min) = w_min · s_xy, which at the median in-plane spacing of this cohort (0.703 mm) is 4.2 mm, but the subpopulation whose strict label is set by the floor is far larger, and the clamped fraction reaches 0.52 at r = 0.6 and 0.72 at r = 0.4 across the whole training set.

### 3.7. Negative Mining

Table 8 reports the negative-mining ablation on the 267-scan cohort common to its configurations. Three of them form the controlled comparison, all with loose adjacent-slice supervision. Restricting background slices to nodule-bearing scans, at the same per-scan count as the full-cohort arm, gave a CPM of 0.8064; matching the total number of background slices while keeping them confined to nodule-bearing scans gave 0.8074; and drawing background slices from all 888 scans gave 0.7758. The two strict configurations in the table are reported for completeness and behave the same way (0.7664 without nodule-free scans against 0.7432 with them).

The three-arm design separates two explanations that a two-arm comparison confounds. The count-matched arm and the reduced-count arm did not differ (paired difference +0.0010, 95% CI −0.0280 to +0.0250, *p* = 0.930), so training-set size does not account for the effect. Drawing background slices from all scans differed from the count-matched arm by −0.0316 (−0.0578 to −0.0015, *p* = 0.040), so the provenance of the background slices does. The direction is contrary to expectation, and so is the mechanism. Against the count-matched arm—the comparison that isolates provenance—drawing background slices from all 888 scans did not suppress spurious responses at all: false positives rose from 14,150 to 17,572 and candidates per scan from 61.7 to 74.7, while upper-bound recall fell from 0.9837 to 0.9728. Systematically including nodule-free scans therefore cost sensitivity without buying specificity on this cohort. Only against the reduced-count arm, which trains on 16% fewer images, does the false-positive count fall (19,694 to 17,572), which is why the count-matched arm is necessary to read the effect correctly.

### 3.8. Multi-Seed Repetition

Table 9 reports three training seeds for the two adjacent-slice configurations over the five-fold sweep cohort. Exp3 gave a mean CPM of 0.8009 with a standard deviation of 0.0168 (range 0.0327) and Exp4 a mean of 0.7591 with a standard deviation of 0.0139 (range 0.0277). The difference between the two configurations (0.0418) exceeds either standard deviation, so the ordering is not attributable to optimisation noise; the seed-to-seed variability is nonetheless of the same order as several of the contrasts examined in the sweeps, which is why those contrasts are reported with paired intervals rather than as point estimates.

### 3.9. Backbone Comparison: YOLO11n and YOLO26n

**Figure 7:**
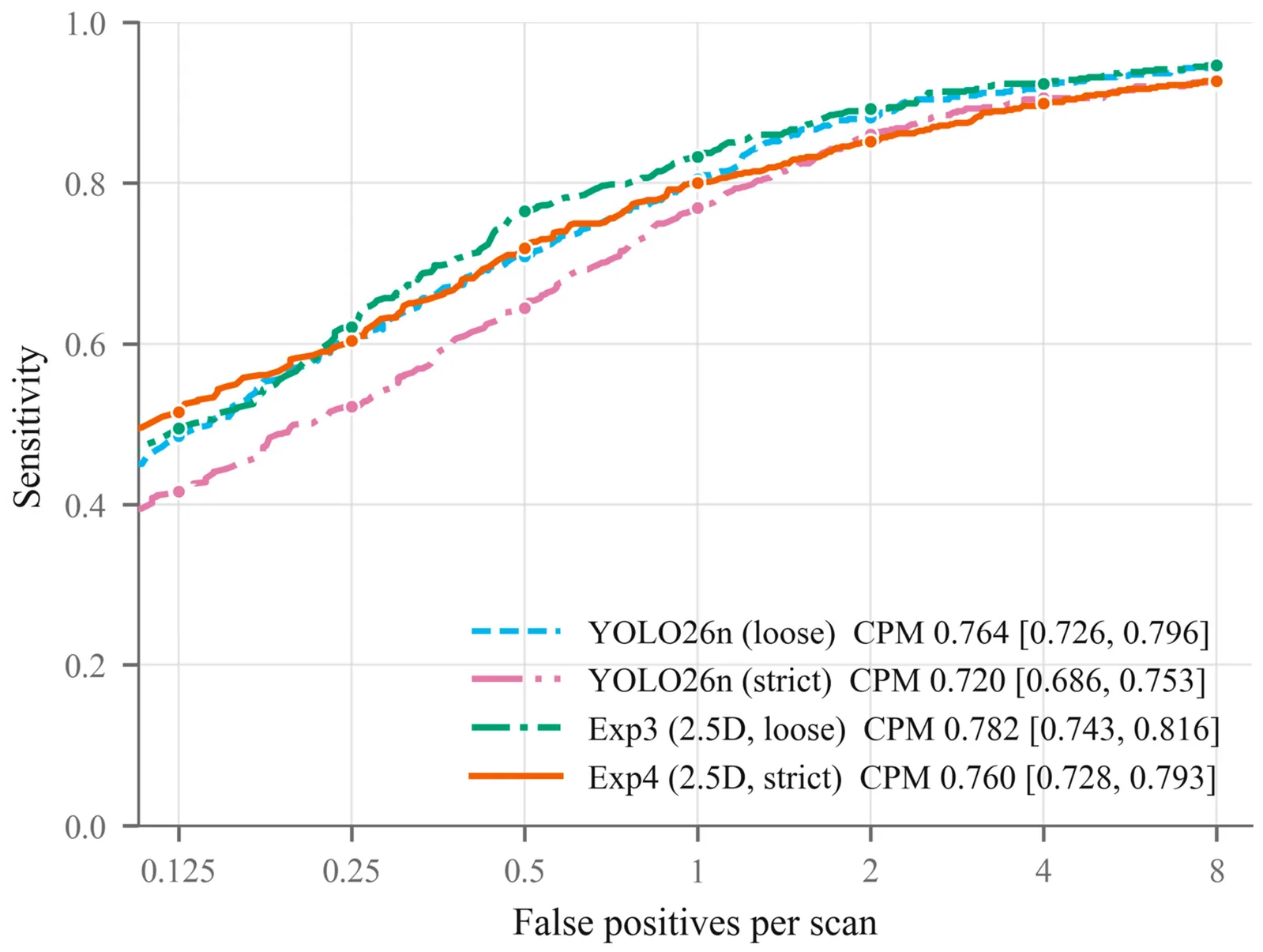
FROC curves for YOLO11n and YOLO26n under matched data, folds, schedule and evaluator, showing that the ordering of the supervision factor is reproduced on a second backbone. Both curves and CPM values are computed on the 445-scan cohort common to the five folds on which the second backbone was trained, so the two YOLO11n values differ slightly from Figure 2, which covers all 888 scans.

Table 10 and Figure 7 compare the two backbones under identical data, folds, schedule and evaluator. On the five-fold cohort, YOLO26n reached 0.7644 with loose supervision and 0.7204 with strict supervision, against 0.7824 and 0.7600 for YOLO11n. The difference between backbones under loose supervision was −0.0179 (95% CI −0.0420 to +0.0058, *p* = 0.148, not significant); under strict supervision YOLO26n was worse by −0.0395 (−0.0636 to −0.0149).

Most relevantly for the present question, the ordering of the supervision factor was reproduced on the second backbone: within YOLO26n, strict supervision was worse than loose by −0.0440 (−0.0721 to −0.0100, *p* = 0.022). The conclusion that core-focused supervision confers no benefit is therefore not specific to one architecture, and in particular survives a detector with an NMS-free end-to-end head and small-target-aware label assignment.

### 3.10. Size-Stratified Recall

Table 11 reports recall by nodule size at a defined operating point of one false positive per scan, rather than over the saturated candidate pool. One false positive per scan is the conventional reporting point for screening computer-aided detection, and reporting at a defined point rather than at saturation is what makes the strata comparable across configurations. At that operating point Exp3 recalled 0.6963 of small (<5 mm), 0.8646 of medium (5–10 mm) and 0.8968 of large (>10 mm) nodules, against 0.4259, 0.6992 and 0.8541 for the 2D baseline. The advantage of the adjacent-slice representation was largest for small and medium nodules (+0.270 and +0.165 recall respectively), which is where through-plane context is most informative for distinguishing a nodule from a vessel cross-section. Exp4 was marginally below Exp3 in all three strata.

### 3.11. Through-Plane Dependence of the Adjacent-Slice Detector

The representation comparison of Section 3.1 establishes that adjacent-slice input improves detection; this section tests the proposed reason. If the advantage arises because the three channels supply through-plane context, then removing that context while leaving the weights and the in-plane appearance untouched should degrade precisely those detections that a 2D detector cannot make.

We therefore replaced the two adjacent-slice channels of each input with copies of the centre slice, reducing the information available to the 2.5D detector to that of a 2D detector without retraining, and recorded the change in detection confidence at the annotated nodule location. The test covers every nodule the adjacent-slice detector finds at a fixed operating point of two false positives per scan: 1049 of the 1186 reference nodules, of which 16 produced no response above 0.01 on the annotated centre slice and are excluded, leaving 1033—divided into those the 2D baseline also detects (*n* = 859) and those only the adjacent-slice detector detects (*n* = 174) (Table 12). The operating point is not a favourable choice: both groups remain well populated across the whole range, the adjacent-slice-only group falling monotonically from 235 nodules at 0.5 false positives per scan to 122 at eight, so the comparison could have been made at any of the seven CPM operating points.

The two groups behave very differently. For nodules that both detectors find, removing the through-plane channels reduced confidence by a median of 6.2%, the group median falling from 0.762 to 0.698: the detection is essentially carried by the central slice. For nodules that only the adjacent-slice detector finds, the same manipulation reduced confidence by a median of 71.3%, the group median falling from 0.599 to 0.119, which in most cases is below any usable operating threshold. The difference between the two distributions of relative reduction is large and highly significant (one-sided Mann–Whitney U, U = 114,922, *p* < 10^−28^, *n* = 1033).

**Figure 8:**
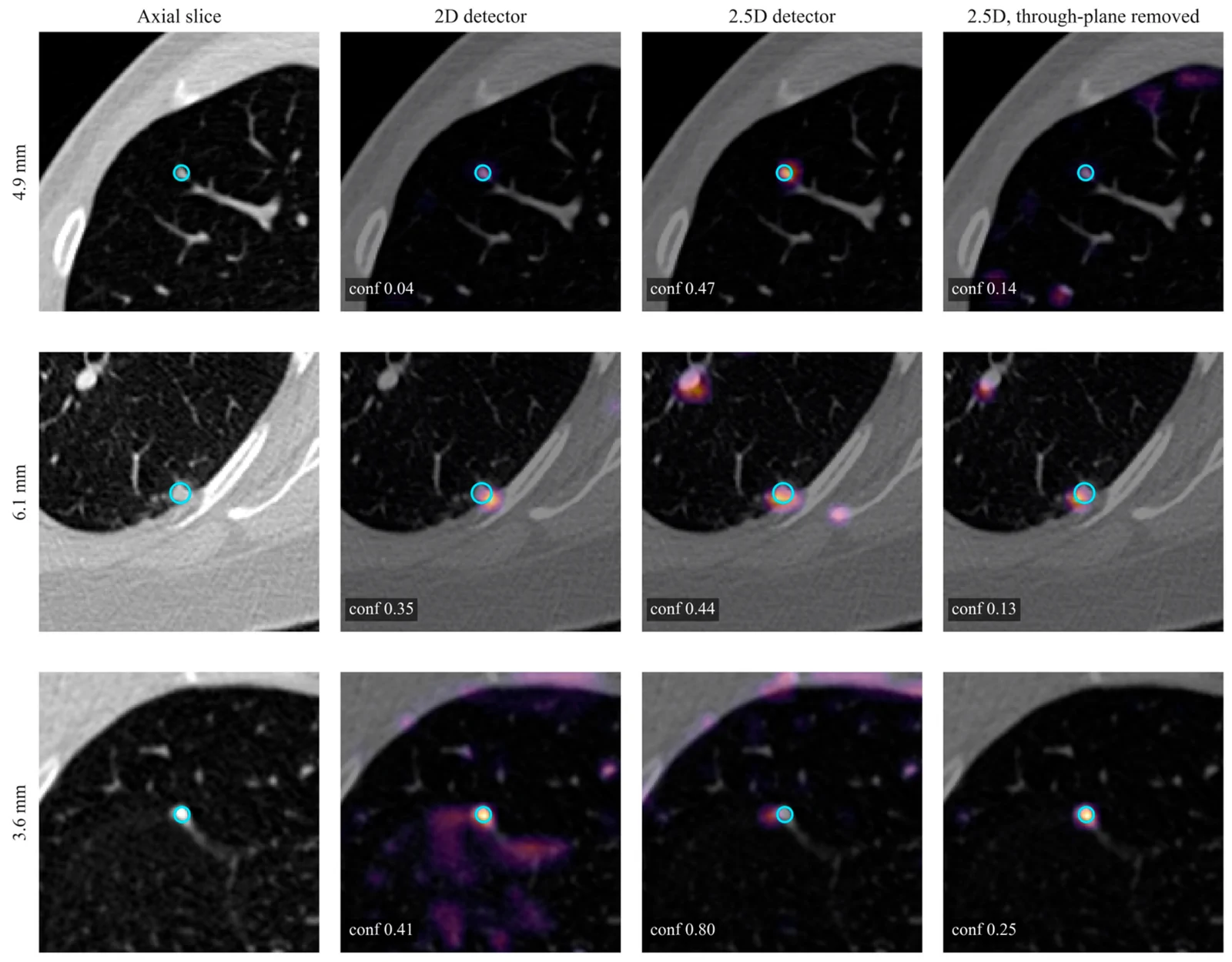
Through-plane dependence, three representative nodules that the 2D detector does not find. Columns: the axial slice; gradient-weighted class activation for the 2D detector; for the adjacent-slice detector; and for the adjacent-slice detector after its two adjacent-slice channels are replaced by copies of the centre slice. Detection confidence at the annotated location is inset, and is the response at the annotated centre slice rather than the aggregated candidate score used for detection; the cyan circle marks the annotated nodule. Attribution is computed at the stride-8 pyramid level, which resolves lesions of this size. Removing the through-plane channels returns the response to the level of the 2D detector, and Table 12 shows that this holds across the cohort.

Figure 8 shows three representative cases, selected as those closest to the median reduction in the second group. In each the 2D detector’s response at the annotated location (0.04, 0.35 and 0.41) falls below the confidence its own two-false-positive operating point requires, 0.43. The adjacent-slice detector responds far more strongly at the same location (0.47, 0.44 and 0.80), and removing the through-plane channels collapses that response to 0.14, 0.13 and 0.25—below its own operating threshold of 0.45 in every case. The values inset in the panels are the response at the annotated centre slice, which is what the ablation manipulates; detection itself uses the aggregated three-dimensional candidate score of Equation (13), so a nodule can be detected on the strength of a neighbouring slice even where the centre-slice response is near the threshold. Gradient-weighted class activation maps [25] are shown for the corresponding predictions, computed at the stride-8 pyramid level, which is the level at which lesions of this size are resolved.

This analysis converts the qualitative observation into a quantitative one: the advantage of the adjacent-slice representation is not a general improvement in feature quality but is specifically attributable to information carried across slices, and it is concentrated on exactly the lesions that a single plane cannot resolve.

### 3.12. Direct Test of the Evaluation Protocol

The comparison between this analysis and the one previously reported changes several things at once, and cannot on its own attribute the changed conclusions to the evaluation protocol. We therefore tested the protocol directly, holding everything else fixed. The checkpoints of Section 3.1, the official folds, the aggregation of Section 2.5, the evaluator and the exclusion list were all left unchanged, and only the set of slices searched was varied: whole-volume inference as reported throughout, against inference restricted to the axial slices containing an annotated nodule centre. Scans with no annotation are then not searched at all, while remaining in the false-positive-per-scan denominator. This is the sharpest form of a reference-standard-selected protocol rather than a replication of any particular one; the earlier pipeline of Section 2.7 also evaluated a small number of sampled background slices, which is why its candidate lists spanned 2.6 slices per scan against 1.32 here. The restricted protocol searches 1176 distinct slices in total—0.52% of the 227,225 searched over whole volumes, a mean of 1.32 per scan across the cohort and 1.96 across the 601 scans that carry an annotation—and yields 1525 to 2062 candidates depending on configuration, against 71,261 to 98,774 over whole volumes (Table 13). That the 1186 annotations occupy only 1176 distinct slices is the same coincidence of centres that produced the label collision described in Section 2.7.

**Figure 9:**
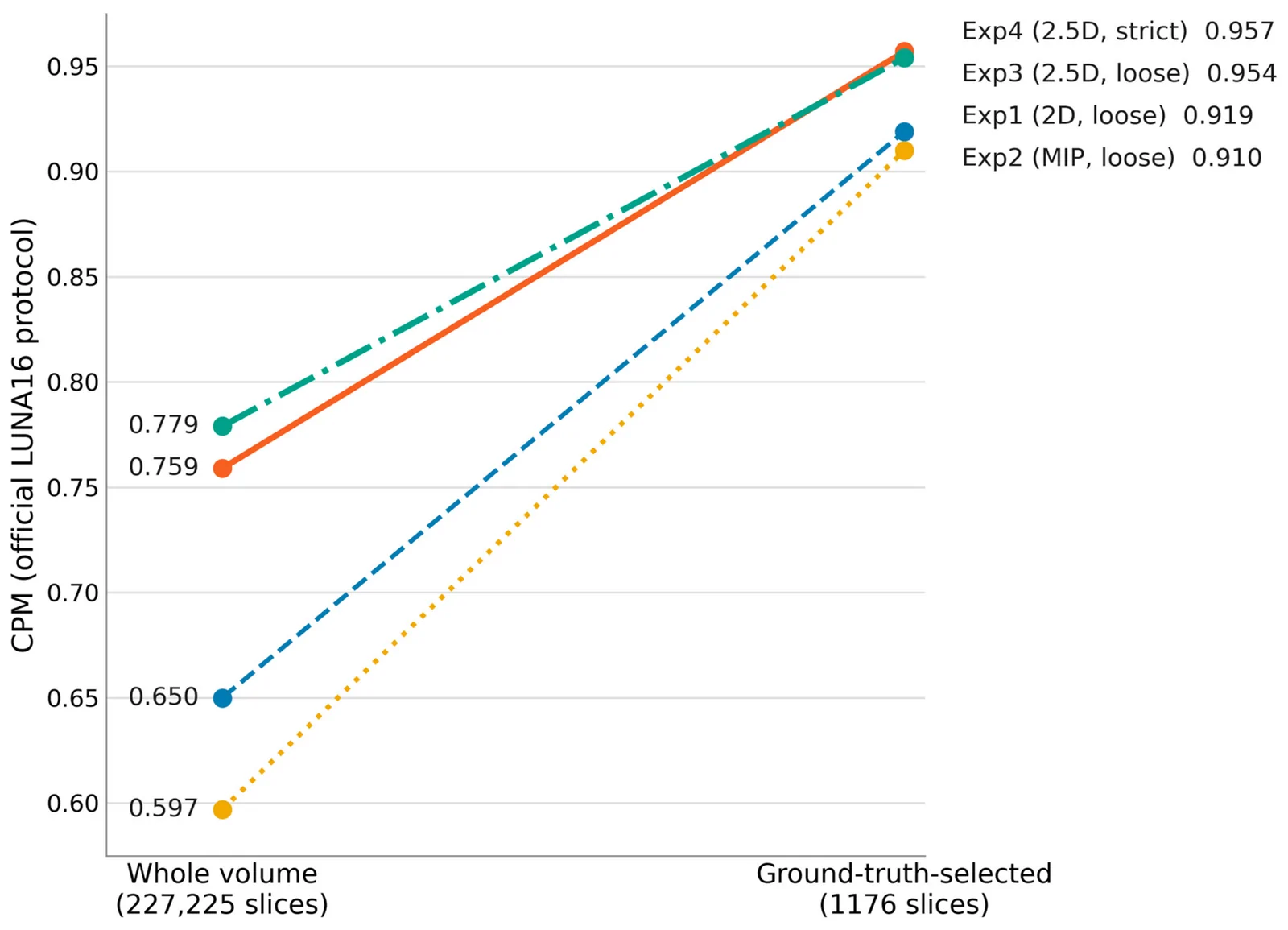
Effect of the evaluation protocol, with the trained models held fixed. Each line joins one configuration’s CPM under whole-volume inference over all 227,225 axial slices of the 888-scan cohort to its CPM when only the 1176 slices containing an annotated nodule centre are searched, 0.52% of the same data; the checkpoints, folds, aggregation, evaluator and exclusion list are identical, so the slice set is the only difference. Every configuration rises and the band compresses, and the two adjacent-slice configurations exchange rank: the supervision contrast reverses (Table 13). Labels are offset vertically with leaders where endpoints coincide.

Restricting the slice set raised CPM for every configuration, by between +0.175 and +0.313, and compressed the spread between configurations from 0.183 (0.5965 to 0.7795) to 0.047 (0.9097 to 0.9568); Figure 9 shows both movements at once. Measured this way, every configuration performs well, because the task has been reduced to describing locations the reference standard has already supplied.

The two contrasts on which this paper’s conclusions rest behave differently under the change, and the distinction matters.

The supervision contrast reverses. Strict core-focused supervision is worse than loose supervision by −0.0209 CPM over whole volumes (95% CI −0.0390 to −0.0008) and better by +0.0027 under the restricted protocol (−0.0086 to +0.0142), where it becomes the top-ranked configuration. The restricted interval spans zero, so that protocol does not merely invert the ordering: it cannot resolve the contrast at all. A study using it would be unable to establish that loose supervision is at least as good.

The representation contrast survives but is strongly attenuated. Adjacent-slice input exceeds the 2D baseline by +0.1297 CPM over whole volumes (+0.1061 to +0.1524) and by +0.0348 under the restricted protocol (+0.0189 to +0.0511)—the same sign, and still excluding zero, but roughly a quarter of the magnitude. The restricted protocol therefore does not conceal the representation effect entirely; it shrinks it to a size at which other sources of error can plausibly bury it, which is consistent with the +0.0003 difference previously reported from a pipeline in which the adjacent-slice baseline was additionally trained on two incomplete folds (Section 2.7).

The conclusion we draw is therefore narrower and better supported than a claim that the protocol inverts everything: the slice set alone reverses the supervision conclusion and attenuates the representation conclusion by roughly a factor of four, while inflating every absolute CPM by 0.17 to 0.31. Both effects follow from the same cause, developed in Section 4.2.

### 3.13. Comparison with Published LUNA16 Results

Table 14 places these results among published LUNA16 detection results, stating the evaluation protocol of each entry. We emphasise that the present system is a single-stage detector with 2.6 million parameters and no false-positive reduction stage, whereas the leading challenge entries are multi-stage systems combining a candidate generator with a separate 3D false-positive reduction network. The purpose of the comparison is to locate this work within the literature and to make the protocol dependence of reported numbers explicit, not to claim competitive ranking.

## 4. Discussion

### 4.1. Principal Findings

Under the official LUNA16 protocol with whole-volume inference, the inter-slice input representation was the dominant training-time design factor in this lightweight detection setting (Section 3.1, Table 1; Section 3.4, Table 4). Adjacent-slice 2.5D stacking exceeded a 2D central-slice baseline by +0.1297 CPM with an effect size of d_z = 2.96 and an advantage in every one of the ten official folds. Thin-slab maximum-intensity projection was worse than a plain 2D slice, indicating that compressing a slab into a single projected plane discards information that the detector can otherwise exploit.

The spatial extent of bounding-box supervision, by contrast, did not improve detection. A six-point ratio sweep found no ratio superior to full-diameter supervision, and the value examined in our earlier analysis, r = 0.6, was not optimal (Section 3.5, Table 6). Across the (r, w_min) design grid, the quantity that tracked performance was neither the ratio nor the minimum box side in isolation, but the fraction of training boxes whose side is fixed by the minimum box constraint rather than by the annotated diameter (Section 3.5, Table 5).

### 4.2. What an Evaluation Restricted to Annotated Slices Measures, and What It Cannot

One of the two conclusions above is the opposite of what our earlier analysis reported, and the other was previously reported as absent. Section 3.12 shows that the evaluation protocol alone accounts for the first and for most of the second, and the asymmetry between them is instructive rather than incidental.

When candidate detections are generated only on slices selected using the reference standard, the detector is asked a different question. It is not asked to find nodules in a volume; it is asked to describe a location it has already been directed to. Two consequences follow directly. First, on the slice containing the nodule centre the 2D and adjacent-slice representations are much closer to equivalent, because the discriminative value of through-plane context lies in rejecting vessel cross-sections that resemble nodules in a single plane—a task that only arises when every slice must be searched. The channel-ablation result of Section 3.11 measures exactly this: for nodules that a 2D detector also finds, removing the through-plane channels costs a median of 6.2% of the detection confidence. The restricted protocol is therefore weighted heavily towards the subpopulation on which the representation matters least, and Section 3.12 quantifies the consequence: the advantage does not disappear, but falls from +0.1297 to +0.0348 CPM. Our earlier analysis reported it as absent altogether (+0.0003); the protocol accounts for three quarters of that gap, and the under-trained adjacent-slice baseline of Section 2.7 is the most likely source of the remainder.

Second, and more consequentially for the supervision factor, such a protocol does not measure whole-volume detection at all. It measures how a detector ranks true against spurious responses within a neighbourhood the reference standard has already identified, and that quantity is close to saturated: under the restricted protocol all four configurations lie between 0.9097 and 0.9568 CPM, a spread of 0.047 against 0.183 over whole volumes (Table 13). Compressed into that band, the supervision contrast not only changes sign (−0.0209 to +0.0027) but ceases to be resolvable at all, its interval spanning zero. We deliberately do not claim that the earlier ranking arose because core-focused supervision produces more false positives; the grid analysis of Section 3.5 shows that the number of false positives does not predict CPM (*p* = 0.68). The defensible statement is narrower and is now measured rather than inferred: the restricted protocol reports a quantity that does not determine whole-volume FROC performance, and for the supervision factor the two disagree in sign.

The same point applies, less dramatically but more pervasively, to the two counting rules of Section 2.1. Applying annotations_excluded.csv raised CPM by between +0.068 and +0.117 here, and the correction is not a constant offset: it differed by 0.049 CPM between the most and least affected configuration and compressed the Exp3-minus-Exp4 contrast by 43% (Section 3.3). The convention adopted for the placeholder diameters inside that list moves CPM by a further 0.034 to 0.050. Each of these is larger than the supervision-extent effect the study set out to measure. A CPM quoted without stating whether the exclusion list was applied, and under which convention, is therefore not comparable with one that does—which is why Table 14 carries a protocol column rather than a bare ranking, and why we report both conventions rather than silently choosing one.

The practical implication is that the protocol is not a neutral container for the comparison. It determined the sign of one effect, the apparent size of the other, and the absolute level of all of them—moving CPM by 0.17 to 0.31, far more than either design factor. We therefore recommend that studies of training-time design factors in nodule detection state explicitly which slices entered inference, state whether the irrelevant-finding list was applied, and treat whole-volume candidate generation as a requirement rather than an implementation detail whenever a per-scan false-positive rate is reported.

### 4.3. Interpretation of the Representation Effect

The magnitude of the representation effect is consistent with the size-stratified results of Section 3.10: the advantage of adjacent-slice input was largest for small and medium nodules, which are precisely the lesions most easily confused with vessel cross-sections in a single axial plane. Three ordered channels allow the detector to observe that a candidate structure appears and disappears over adjacent slices, or alternatively persists as a tubular structure, without any 3D convolution.

That thin-slab MIP performed worse than a plain 2D slice is consistent with the same account. A maximum-intensity projection retains the brightest voxel along the through-plane axis and discards the ordering; it therefore removes the very cue that adjacent-slice stacking supplies, while additionally superimposing high-attenuation vessels onto the plane, which increases the density of nodule-like appearances. Our earlier analysis, which found MIP broadly comparable to the other configurations, was again measuring on annotated slices where this cue is not required.

The channel-ablation analysis of Section 3.11 supports this account directly rather than by inference. Detections that a 2D detector can also make survive the removal of the adjacent-slice channels almost unchanged, losing a median 6.2% of their confidence, whereas detections unique to the adjacent-slice detector lose a median 71.3% and mostly fall below any usable threshold. The representation therefore does not improve the detector generally; it supplies a specific cue that is decisive for a specific subpopulation of lesions, which is why the size-stratified results show the largest gains for small and medium nodules and why an evaluation confined to the annotated central slice cannot detect the effect.

### 4.4. Supervision Extent, the Minimum Box Constraint, and What Should Be Reported

The supervision-extent factor (Section 3.5 and Section 3.6; Table 5, Table 6 and Table 7) requires a more careful statement than the one we previously offered. Reducing the training box does not simply concentrate supervision on the lesion core, because Equation (9) is piecewise: for a lesion whose chord diameter falls below d × (r, w_min) the box side is set by the floor, so the ratio does not scale that box at all. The proportion of the training set in that regime is substantial at the ratios typically used—52% at r = 0.6 with a six-pixel floor in this cohort—so a sweep over r is partly a sweep over the clamped fraction.

The minimum box sweep separates them, and its outcome was not the one we anticipated. The intuitive account—smaller boxes yield more proposals, and whole-volume scanning charges for each—is not supported by these data: across the design grid the number of false positives was uncorrelated with CPM (*p* = 0.68), as was the number of candidates per scan (*p* = 0.74). This is expected on reflection, since FROC consults only the highest-scoring candidates and surplus low-scoring proposals are invisible to it. What tracked performance was the clamped fraction (Pearson r = −0.88, *p* = 0.004), that is, the proportion of the training signal in which label size no longer varies with lesion size.

We therefore suggest that the operative variable is the geometric fidelity of the supervision rather than its extent: labels that continue to track lesion size train a better detector than labels that do not, whether fidelity is lost by shrinking the ratio or by raising the floor. This does not contradict the radiomic evidence that the tissue immediately surrounding a nodule carries diagnostic information [13,14]; it separates two questions. That literature concerns characterisation, where the lesion has already been localised and the perinodular region is an additional source of signal. Detection asks something else—where to look—and there the value of a label lies in how faithfully its extent reports lesion size, which is the quantity the clamped fraction measures. This yields a concrete recommendation. A minimum box size is imposed in most detection pipelines to keep label assignment stable and is seldom reported; here it exerted an influence comparable to the factor under study. Studies sweeping a supervision ratio should report the clamped fraction alongside it, since otherwise two quantities are varied at once.

### 4.5. Negative Mining and the Minimum-Box Constraint

The negative-mining result (Section 3.7, Table 8) was also contrary to expectation, in outcome and in mechanism. Systematically drawing background slices from all 888 scans, including the 287 that contain no reference nodule, lowered CPM, and against the count-matched arm it did not even buy the specificity such sampling is meant to buy, since false positives and candidates per scan both rose. The count-matched arm establishes that neither effect is an artefact of training-set size.

We do not have a controlled explanation for the direction of this effect, and we present it as an empirical observation to be examined rather than as a recommendation against negative mining. One possibility is that nodule-free LUNA16 scans do not have an anatomically neutral background: they are scans in which no finding attained four-radiologist consensus, yet they still contain abundant structures listed among the irrelevant findings, so training on them supplies negatives whose appearance overlaps that of the positives, which can blur the decision boundary rather than sharpen it. Distinguishing this from a simpler change in the effective positive fraction would require sampling designs that hold the positive fraction fixed while varying provenance, which we identify as future work.

### 4.6. Limitations

Overfitting and optimisation noise. Model selection used a validation subset disjoint from both training and test partitions, removing the selection bias present in our earlier analysis. Run-to-run variability was measured rather than assumed: across three seeds the standard deviation of CPM was 0.0168 for Exp3 and 0.0139 for Exp4 (Section 3.8, Table 9). That variability is comparable to several of the sweep contrasts, and those contrasts are correspondingly reported with paired intervals rather than as point estimates.

Dependence on data partitioning. The scan-level and fold-wise analyses (Section 3.1 and Section 3.4, Table 4) answer different questions and, for the supervision-extent contrast, give different answers. We report both. Where they disagree we adopt the more conservative reading, and no claim in this paper rests on a contrast that is significant at the scan level but not fold-wise.

Domain shift. LUNA16 is a single curated cohort—scans thicker than 2.5 mm were excluded when it was assembled, and the slice spacing of the 888 scans ranges from 0.45 to 2.50 mm with a median of 1.25 mm—with a consensus reference standard from four radiologists. Scanner models, reconstruction kernels, dose protocols, slice spacing and annotation conventions all differ in deployment, and each can shift the operating point of a detector trained as described here.

Absence of external validation. No independent cohort was evaluated. Nothing in these results licences a statement about performance on data acquired elsewhere, and we regard multi-centre external validation as a precondition for any clinical claim rather than as an optional extension.

Over-interpretation of a single public benchmark. Our own earlier analysis is the cautionary case: a self-consistent set of results on a widely used public dataset supported a conclusion that a change in protocol reversed. Section 3.12 shows that this is not hindsight—with the models held fixed, the slice set alone flips the supervision contrast and inflates every CPM by 0.17 to 0.31. Absolute CPM values on LUNA16 are meaningful only relative to a stated protocol, and a ranking between design choices can invert when that protocol changes.

Scope. The comparison covers two lightweight anchor-free detectors at one input resolution, a single-stage pipeline without a false-positive reduction network, and one supervision family. The qualitative and quantitative results should not be extrapolated to full 3D convolutional detectors, transformer-based architectures or multi-stage computer-aided detection (CAD) systems without direct evaluation.

## 5. Conclusions

We benchmarked two training-time design choices for lightweight 2.5D pulmonary nodule detection under the official LUNA16 subset0–subset9 protocol, with whole-volume inference over all 888 scans, the official irrelevant-finding exclusion list, and validation partitions disjoint from the test partitions.

The inter-slice input representation was the dominant factor. Adjacent-slice 2.5D stacking exceeded a 2D central-slice baseline by +0.1297 CPM (0.7795 versus 0.6498) with an advantage in all ten official folds, while thin-slab maximum-intensity projection was inferior to a plain 2D slice. Reducing the spatial extent of bounding-box supervision conferred no benefit: a six-point ratio sweep identified no ratio superior to full-diameter supervision, and across the design grid performance tracked the fraction of training boxes clamped at the minimum box size more closely than it tracked the supervision ratio itself.

Re-scoring the same checkpoints over only the slices that contain an annotated nodule centre changes these findings in two different ways. The supervision conclusion reverses: core-focused supervision becomes the top-ranked configuration, and the contrast ceases to be resolvable at all. The representation conclusion survives with the same sign but falls to roughly a quarter of its magnitude, because the discriminative work of through-plane context lies in rejecting structures on slices that such a protocol never examines. Every absolute CPM rises by 0.17 to 0.31. We therefore report the protocol dependence itself as a result, established with the trained models held fixed: in this design space the slice set searched at inference decides the sign of one factor, the apparent size of the other, and the absolute level of both.

For practitioners developing lightweight detectors for resource-constrained screening workflows, the actionable findings are that adjacent-slice stacking is worth its negligible cost and should be preferred to projection-based slab compression, that shrinking supervision boxes is not a productive avenue, and that the minimum box size imposed during label generation should be reported because it influences the outcome as much as the factor under study. Complete code, weights, candidate lists and a verified re-implementation of the official evaluation script are released so that each of these statements can be checked and re-scored independently.

## Figures and Tables

**Table 1:** Competition performance metric (CPM) for the four configurations under the official LUNA16 subset0–subset9 ten-fold protocol, evaluated over all 888 scans and 1186 annotated nodules with whole-volume inference and the official irrelevant-finding exclusion list applied. Confidence intervals are percentile intervals from 1000 scan-level bootstrap resamples. Upper-bound recall is the proportion of nodules represented in the retained candidate pool. Best value per column in bold.

Configuration	Input Representation	Supervision	CPM	95% CI	Candidates/Scan	False Positives	Upper-Bound Recall
Exp1	2D central slice	Loose (r = 1.0)	0.6498	0.6181–0.6778	111.2	90,782	0.9503
Exp2	Thin-slab MIP	Loose (r = 1.0)	0.5965	0.5703–0.6239	93.1	75,060	0.9030
Exp3	Adjacent-slice 2.5D	Loose (r = 1.0)	**0.7795**	0.7517–0.8005	80.2	63,159	0.9755
Exp4	Adjacent-slice 2.5D	Strict (r = 0.6)	0.7586	0.7332–0.7821	83.0	65,235	0.9680

**Table 2:** Sensitivity at each of the seven CPM operating points, official protocol, 888-scan denominator. Every candidate pool extends past 8 FP/scan, so no value is extrapolated.

Configuration	0.125	0.25	0.5	1	2	4	8	CPM
Exp1	0.3562	0.4696	0.5896	0.6721	0.7648	0.8297	0.8668	0.6498
Exp2	0.2962	0.4060	0.5304	0.6304	0.7155	0.7793	0.8179	0.5965
Exp3	0.4921	0.6265	0.7516	0.8334	0.8845	0.9233	0.9452	0.7795
Exp4	0.5037	0.6019	0.7229	0.7993	0.8562	0.9022	0.9241	0.7586

**Table 3:** All 888 scans. CPM computed without the irrelevant-finding exclusion list and with it. Candidates falling inside an irrelevant finding are ignored under the official rule rather than counted as false positives. The final column applies the convention under which the archive’s reference output was generated, in which the placeholder diameters of −1 mm are taken at their absolute value instead of the 10 mm fallback used by the script as currently distributed (Section 2.1); it is reported because the choice moves CPM by more than the difference between the two supervision settings.

Configuration	CPM, No Exclusion List	CPM, Exclusion List (10 mm Fallback)	Δ CPM	Candidates Ignored	FP Removed	CPM, Pre-Fallback Convention
Exp1	0.5675	0.6498	+0.0824	6387	6.57%	0.6163
Exp2	0.5288	0.5965	+0.0677	5939	7.33%	0.5586
Exp3	0.6779	0.7795	+0.1016	6361	9.15%	0.7304
Exp4	0.6414	0.7586	+0.1172	6744	9.37%	0.7089

**Table 4:** All 888 scans, scored within each of the ten official test subsets (88–89 scans each). Paired comparisons are across the ten folds; *p*-values are Holm–Bonferroni adjusted across the family of primary contrasts, and d_z is the paired effect size.

**Test Subset**		**Exp1**		**Exp2**		**Exp3**		**Exp4**	
subset0		0.5816		0.5644		0.7695		0.7541	
subset1		0.7344		0.6320		0.8020		0.7301	
subset2		0.7176		0.6585		0.8121		0.7479	
subset3		0.6351		0.5954		0.7716		0.7833	
subset4		0.7515		0.6177		0.8073		0.7924	
subset5		0.5900		0.5731		0.7536		0.7636	
subset6		0.6336		0.6368		0.7808		0.7398	
subset7		0.6633		0.6005		0.7925		0.8168	
subset8		0.6392		0.5266		0.7482		0.6622	
subset9		0.6626		0.5660		0.8054		0.8023	
**Mean**		**0.6609**		**0.5971**		**0.7843**		**0.7592**	
**Contrast**	**Folds Favouring First**		**Mean Difference**		**Median**		**Wilcoxon *p* (Holm)**		**d_z**
Exp4 vs. Exp3	3/10		−0.0251		−0.0151		0.1055		−0.65
Exp4 vs. Exp1	9/10		+0.0983		+0.1229		0.0117		+1.43
Exp3 vs. Exp1	10/10		+0.1234		+0.1328		0.0098		+2.96
Exp3 vs. Exp2	10/10		+0.1872		+0.1851		0.0098		+6.40
Exp1 vs. Exp2	9/10		+0.0638		+0.0609		0.0117		+1.38

Bold indicates the best value in each column.

**Table 5:** Every cell of the (r, w_min) grid evaluated on the 445-scan cohort common to the sweep configurations, ordered by the clamped fraction ρ of Equation (11)—the proportion of training boxes whose side is set by the minimum box constraint rather than by the annotated diameter. Correlation of each candidate predictor with CPM across the eight grid cells.

**r**	**w_min (px)**		**Clamped Fraction ρ**		**CPM**		**False Positives**		**Upper-Bound Recall**
1	6		0.186		0.7824		32,421		0.9805
0.6	4		0.249		0.7636		36,169		0.9854
0.8	6		0.329		0.7604		36,486		0.9789
0.7	6		0.425		0.7622		34,093		0.9691
0.6	6		0.520		0.7600		36,074		0.9740
0.5	6		0.620		0.7225		33,292		0.9707
0.6	8		0.671		0.7253		33,966		0.9642
0.4	6		0.723		0.7373		34,043		0.9659
**Predictor**		**Pearson r**		***p***		**Spearman ρ**		***p***	
Clamped fraction ρ		−0.880		0.0039		−0.881		0.0039	
Supervision ratio r		+0.765		0.0268		+0.732		0.0390	
Upper-bound recall		+0.725		0.0418		+0.714		0.0465	
Minimum box side		−0.487		0.2213		−0.545		0.1619	
False positives		+0.173		0.6819		+0.214		0.6103	
Candidates per scan		+0.139		0.7437		+0.143		0.7358	

**Table 6:** CPM against the supervision-extent ratio, 445-scan common cohort. Paired differences are relative to r = 0.6.

**r**	**CPM**			**95% CI**		
0.4	0.7373			0.7013–0.7716		
0.5	0.7225			0.6826–0.7549		
0.6	0.7600			0.7277–0.7934		
0.7	0.7622			0.7254–0.7961		
0.8	0.7604			0.7216–0.7922		
1	0.7824			0.7427–0.8161		
**Contrast**		**Δ CPM**	**95% CI**		***p***	**Excludes Zero**
r = 0.4 vs. r = 0.6		−0.0227	−0.0448 to −0.0041		0.014	yes
r = 0.5 vs. r = 0.6		−0.0375	−0.0638 to −0.0183		0.002	yes
r = 0.7 vs. r = 0.6		+0.0022	−0.0284 to +0.0253		0.912	no
r = 0.8 vs. r = 0.6		+0.0005	−0.0297 to +0.0209		0.880	no
r = 1.0 vs. r = 0.6		+0.0224	−0.0091 to +0.0476		0.152	no

**Table 7:** CPM against the minimum box side at fixed supervision ratio, 445-scan common cohort. Paired differences are relative to w_min = 6 px.

**w_min (px)**		**CPM**			**95% CI**	
4		0.7636			0.7311–0.7971	
6		0.7600			0.7277–0.7934	
8		0.7253			0.6885–0.7618	
**Contrast**	**Δ CPM**		**95% CI**	***p***		**Excludes Zero**
w_min = 4 px vs. w_min = 6 px	+0.0037		−0.0196 to +0.0271	0.706		no
w_min = 8 px vs. w_min = 6 px	−0.0347		−0.0587 to −0.0104	0.010		yes

**Table 8:** Configurations differing in the provenance and count of background training slices, on the 267-scan cohort common to them. The three loose (2.5D) arms form the controlled comparison: background slices drawn from all 888 scans, the same per-scan count drawn from nodule-bearing scans only, and a third arm in which the total number of background slices is matched to the first while still confined to nodule-bearing scans. The third arm separates the effect of scan provenance from that of training-set size. The two strict (r = 0.6) rows are reported for completeness and are not part of that comparison.

**Arm**	**Background Slices Drawn from**	**Mean Training Images**		**CPM**		**95% CI**		**False Positives**	
2.5D loose (Exp3)	All 888 scans	11,418		0.7758		0.7213–0.8288		17,572	
2.5D loose, reduced negative count	Nodule-bearing scans only	9582		0.8064		0.7641–0.8489		19,694	
2.5D loose, negative count matched	Nodule-bearing scans only	11,342		0.8074		0.7531–0.8528		14,150	
2.5D strict (Exp4)	All 888 scans	11,418		0.7432		0.6944–0.7849		21,566	
2.5D strict, reduced negative count	Nodule-bearing scans only	9582		0.7664		0.7136–0.8093		20,124	
**Contrast**			**Δ CPM**		**95% CI**		***p***		**Excludes Zero**
2.5D loose, negatives from all 888 scans vs. 2.5D loose, nodule-bearing only, count-matched			−0.0316		−0.0578 to −0.0015		0.040		yes
2.5D loose, nodule-bearing only, count-matched vs. 2.5D loose, nodule-bearing scans only			+0.0010		−0.0280 to +0.0250		0.930		no
2.5D loose, negatives from all 888 scans vs. 2.5D loose, nodule-bearing scans only			−0.0306		−0.0608 to −0.0012		0.034		yes
2.5D strict, negatives from all 888 scans vs. 2.5D strict, nodule-bearing scans only			−0.0232		−0.0493 to +0.0053		0.078		no

**Table 9:** Three training seeds (42, 1337, 2026) for the two adjacent-slice configurations, on the 445-scan cohort of the five-fold sensitivity analyses.

Configuration	Seeds	Mean CPM	SD	Min	Max	Range
Exp3 (2.5D loose)	3	0.8009	0.0168	0.7824	0.8151	0.0327
Exp4 (2.5D strict)	3	0.7591	0.0139	0.7448	0.7725	0.0277

**Table 10:** Identical data, folds, training schedule and evaluator; only the backbone differs. On the 445-scan common cohort.

**Backbone**	**Supervision**			**CPM**		**95% CI**	
yolo26n	Loose (r = 1.0)			0.7644		0.7256–0.7964	
yolo26n	Strict (r = 0.6)			0.7204		0.6856–0.7531	
yolo11n	Loose (r = 1.0)			0.7824		0.7427–0.8161	
yolo11n	Strict (r = 0.6)			0.7600		0.7277–0.7934	
**Contrast**		**Δ CPM**	**95% CI**		***p***		**Excludes Zero**
YOLO26n, strict vs. YOLO11n, strict		−0.0395	−0.0636 to −0.0149		0.000		yes
YOLO26n, loose vs. YOLO11n, loose		−0.0179	−0.0420 to +0.0058		0.148		no
YOLO26n, strict vs. YOLO26n, loose		−0.0440	−0.0721 to −0.0100		0.022		yes

**Table 11:** All 888 scans and 1186 nodules. Recall by annotated nodule diameter at a defined operating point of 1 FP/scan, rather than over the saturated candidate pool, which reports coverage at an unusably permissive threshold.

Size Group	Nodules	Exp1	Exp2	Exp3	Exp4
small (<5 mm)	270	0.4259	0.4259	0.6963	0.6852
medium (5–10 mm)	635	0.6992	0.6409	0.8646	0.8346
large (>10 mm)	281	0.8541	0.8149	0.8968	0.8292

**Table 12:** Detection confidence at the annotated nodule location with the intact 2.5D input and with the two adjacent-slice channels replaced by copies of the centre slice, which removes through-plane information without retraining and without altering the in-plane appearance. Nodules are taken at an operating point of 2 false positives per scan and grouped by whether the 2D baseline also detects them. All 1049 nodules that the adjacent-slice detector detects at this operating point were evaluated; 16 produced no response above 0.01 on the annotated centre slice, where the ablation is applied, and are excluded. The confidence columns are group medians; the final column is the median of the per-nodule relative reductions, which is the quantity the test is applied to and is therefore not the ratio of the two preceding columns. One-sided Mann–Whitney U test on the relative reduction (adjacent-slice-only greater than both): *p* < 0.0001.

Nodule Group	*n*	Confidence, Intact Input	Confidence, Through-Plane Removed	Relative Reduction
Detected by both detectors	859	0.762	0.698	6.2%
Detected only by the adjacent-slice detector	174	0.599	0.119	71.3%

**Table 13:** The same checkpoints, folds, aggregation, evaluator and exclusion list as Table 1, scored twice: over every axial slice, and over only the slices that contain an annotated nodule centre. Scans without an annotation are not searched at all and still count in the false-positive-per-scan denominator. The slice set is the only difference between the two columns, so the change is attributable to it alone. The restricted protocol searches 1176 distinct slices, 0.52% of the 227,225 searched over whole volumes. All 888 scans and 1186 nodules. Paired bootstrap contrasts under each protocol, on resamples shared by all four configurations within that protocol.

**Configuration**	**CPM, Whole Volume**		**CPM, GT-Selected Slices**		**Δ**	**Candidates, Whole Volume**		**Candidates, GT-Selected**		**Rank, Whole Volume**		**Rank, GT-Selected**
Exp1	0.6498		0.9193		+0.2695	98,774		1940		3		3
Exp2	0.5965		0.9097		+0.3131	82,651		2062		4		4
Exp3	0.7795		0.9541		+0.1746	71,261		1618		1		2
Exp4	0.7586		0.9568		+0.1981	73,738		1525		2		1
**Contrast**		**Δ CPM, Whole Volume**		**95% CI**			**Δ CPM, GT-Selected**		**95% CI**		**Sign**	
representation (2.5D–2D)		+0.1297		+0.1061 to +0.1524			+0.0348		+0.0189 to +0.0511		preserved	
supervision (strict–loose)		−0.0209		−0.0390 to −0.0008			+0.0027		−0.0086 to +0.0142		**reverses**	

Bold indicates the best value in each column.

**Table 14:** Reported performance is comparable only within a protocol: the official subset partition, whole-volume candidate generation and the official irrelevant-finding exclusion list each affect the number obtained. The final column states whether an entry is commensurable with the CPM reported here; entries evaluated on random slice partitions, or reporting mean average precision rather than FROC-based CPM, are not. Entries without a citation are challenge leaderboard submissions, for which the organisers published a score but the participants did not publish a method description; they are listed from the official leaderboard.

Method	Year	Architecture	Evaluation Protocol	Metric	Value	Commensurable
PAtech	2016	undisclosed complete CAD system	official LUNA16 detection track	CPM	0.951	yes
JianPeiCAD	2016	undisclosed complete CAD system	official LUNA16 detection track	CPM	0.95	yes
LUNA16FONOVACAD	2016	undisclosed complete CAD system	official LUNA16 detection track	CPM	0.947	yes
iFLYTEK-MIG	2016	undisclosed complete CAD system	official LUNA16 detection track	CPM	0.941	yes
zhongliu_xie	2016	undisclosed complete CAD system	official LUNA16 detection track	CPM	0.922	yes
iDST-VC	2016	undisclosed complete CAD system	official LUNA16 detection track	CPM	0.897	yes
qfpxfd	2016	undisclosed complete CAD system	official LUNA16 detection track	CPM	0.891	yes
CASED	2017	3D CNN curriculum adaptive sampling	official LUNA16 detection track	CPM	0.887	yes
3DCNN_NDET	2016	3D CNN	official LUNA16 detection track	CPM	0.882	yes
Aidence	2016	undisclosed complete CAD system	official LUNA16 detection track	CPM	0.871	yes
Ding et al. [5]	2017	2D Faster R-CNN candidate generation + 3D DCNN false-positive reduction	official 10-fold subset CV (as reported)	CPM	0.891	yes
DeepLung (Zhu et al.) [6]	2018	3D dual-path Faster R-CNN	official 10-fold subset CV (as reported)	CPM	0.842	yes
WTAM-YOLO (Lan et al.) [7]	2026	YOLOv11 + wavelet conv + CBAM + HRAMi + iEMA	random 8:1:1 split of augmented 2D slices	mAP@50	0.931	no
**This work (Exp3)**	2026	YOLO11n lightweight 2.5D, single stage, loose (r = 1.0) (2.6 M parameters)	official subset0–9 ten-fold CV, whole-volume inference, annotations_excluded applied	CPM	**0.7795**	yes
**This work (Exp4)**	2026	YOLO11n lightweight 2.5D, single stage, strict (r = 0.6) (2.6 M parameters)	official subset0–9 ten-fold CV, whole-volume inference, annotations_excluded applied	CPM	**0.7586**	yes

Bold indicates the best value in each column.

## Data Availability

The original contributions presented in this study are included in the article/Supplementary Material. Further inquiries can be directed to the corresponding author. The LUNA16 dataset analysed in this study is publicly available at https://luna16.grand-challenge.org/ (accessed on 1 June 2025). The underlying imaging data are derived from the LIDC-IDRI collection [17] hosted by The Cancer Imaging Archive [18]. The official evaluation archive, including annotations.csv, annotations_excluded.csv, seriesuids.csv and the reference evaluation script, was obtained from Zenodo [19] (doi:10.5281/zenodo.3723295). All code, trained weights for every configuration and fold, the full-volume candidate lists, the verified Python-3 re-implementation of the official evaluation script with its automated verification test, the false-positive composition table and ranked false-positive lists of Section 3.3, and every result table and figure reported here are publicly available under a Creative Commons Attribution 4.0 International (CC-BY-4.0) licence at https://huggingface.co/Lien-Feng/Lightweight-2-5D-LUNA16 (accessed on 3 September 2026). The exact version underlying the results reported here is archived under a persistent identifier (DOI: 10.57967/hf/10295, https://doi.org/10.57967/hf/10295; repository commit d9bc7ec3f06fab778f941fd8f05de145f1a7332c).

## Supplementary Materials

The following supporting information can be downloaded at https://www.mdpi.com/article/10.3390/diagnostics16183038/s1. Supplementary File S1, Mathematical Framework.
