# Supplementary material for: Inter-Slice Representation Outweighs Bounding-Box Supervision Extent in Lightweight 2.5D Pulmonary Nodule Detection: A Whole-Volume Benchmark on LUNA16
Source: Diagnostics (Basel). 2026 Sep 19;16(18):3038. doi: 10.3390/diagnostics16183038 (PMC13605363; doi:10.3390/diagnostics16183038)
Supplement: Supplementary file 1 [file diagnostics-16-03038-s001.zip › diagnostics-4441850-supplementary.pdf]

Supplementary File S1. Mathematical Framework

Manuscript ID: diagnostics-4441850 · *Diagnostics* (MDPI)

Title: Inter-Slice Representation Outweighs Bounding-Box Supervision Extent in Lightweight 2.5D Pulmonary Nodule Detection: A Whole-Volume Benchmark on LUNA16

Authors: Lien-Feng Chou, Bing-Ru Peng, Shou-Wei Chien, Yu-Ming Huang

This document provides the complete derivation of the mathematical framework summarised in Section 2 of the main text. It gives Equations (1)–(15) together with the intermediate steps that a Methods section has no room for. The notation is self-contained: every symbol used in the Results is defined here, and each equation carries the number it has in the paper.

Equations	Manuscript section	Content
(1)-(4)	2.1	hit criterion, candidate partition, FROC and CPM
(5)-(7)	2.2, 2.3	voxel-to-world map, intensity window, the three channel operators
(8)-(11)	2.3	spherical-chord supervision, the box-side rule, the clamping regime
(12)-(14)	2.5	slice detections to 3D candidates, and the inverse coordinate map
(15)	2.6	paired scan-level bootstrap

A. Scoring: hits, irrelevant findings, FROC and CPM *(Section 2.1)*

Let $\mathcal{S}$ be the evaluation cohort ($|\mathcal{S}| = 888$), $\mathcal{G}$ the reference nodules ($|\mathcal{G}| = 1186$) and $\mathcal{E}$ the irrelevant findings of `annotations_excluded.csv`. A candidate c **hits** an annotation a when its centre falls inside the annotated ball,

$$\text{hit}(c, a) \;\Leftrightarrow \| \mathbf{x}_c - \mathbf{x}_a \|_2^2 < \;(d_a/2)^2, \qquad d_a \leftarrow 10 \text{ mm if } d_a < 0.$$

The substitution in (1) matters mainly for $\mathcal{E}$: 30,513 of the 35,192 irrelevant findings carry a placeholder diameter of -1, and `noduleCADEvaluationLUNA16.py` as currently distributed replaces a negative diameter with 10 mm. Every result reported in the paper uses that rule. The `CADAnalysis.txt` reference bundled with the same archive predates it, so reproducing that file requires $d_a \leftarrow |d_a|$ instead; `luna_rev.evaluate` implements both (`excluded_policy="official"` and `"legacy_abs"`) and Table 3 of the paper reports CPM under each, because the choice moves CPM by 0.034-0.050 - more than the difference between the two supervision settings.

Each scan's candidates are partitioned in this order:

$$\mathcal{C} = \underbrace{\mathcal{C}^{\mathrm{TP}}}_{\text{one per hit } g \in \mathcal{G}} \sqcup \underbrace{\mathcal{C}^{\mathrm{dup}}}_{\text{further hits on the same } g} \sqcup \underbrace{\mathcal{C}^{\mathrm{irr}}}_{\text{hits on } \mathcal{E}} \sqcup \underbrace{\mathcal{C}^{\mathrm{FP}}}_{\text{remainder}} .$$

$\mathcal{C}^{\mathrm{TP}}$ takes the highest-scoring hit, $q_g = \max\{p_c : \mathrm{hit}(c, g)\}$. $\mathcal{C}^{\mathrm{dup}}$ **and** $\mathcal{C}^{\mathrm{irr}}$ **are discarded, not scored** - this is precisely the role of `annotations_excluded.csv`, and omitting it inflates the false-positive count by $|\mathcal{C}^{\mathrm{irr}}|$.

Sweeping a threshold t over the retained scores gives

$$\mathrm{Sens}(t) = \frac{|\{g \in \mathcal{G} : g \text{ detected},\ q_g \geq t\}|}{|\mathcal{G}|}, \qquad \mathrm{FP/scan}(t) = \frac{|\{c \in \mathcal{C}^{\mathrm{FP}} : p_c \geq t\}|}{|\mathcal{S}|},$$

and eliminating t yields the FROC curve $\mathcal{F}$: FP/scan $\mapsto$ Sens. The competition performance metric is that curve sampled at seven fixed operating points,

$$\boxed{\mathrm{CPM} = \frac{1}{7} \sum_{\eta \in H} \mathcal{F}(\eta), \qquad H = \{1/8, 1/4, 1/2, 1,2,4,8\}}$$

with $\mathcal{F}$ linearly interpolated at each η. Note that (3) divides by $|\mathcal{S}| = 888$ **whether or not a scan produced candidates**, so whole-volume inference over all 888 scans - including the 287 with no reference nodule - is a precondition for (4) to mean what it says.

B. Volume, intensity normalisation and slice operators *(Section 2.2-2.3)*

A CT study is a scalar field sampled on a voxel lattice. Write a scan as

$$V : \Omega \subset \mathbb{Z}^3 \to \mathbb{R}, \qquad \Omega = \{0, \ldots, N_x - 1\} \times \{0, \ldots, N_y - 1\} \times \{0, \ldots, N_z - 1\},$$

with voxel spacing $\mathbf{s} = (s_x, s_y, s_z)$ in millimetres, origin $\mathbf{o} \in \mathbb{R}^3$ and direction cosine matrix $D \in \mathbb{R}^{3\times 3}$. Voxel indices and world coordinates are related by the affine map

$$\phi(\mathbf{v}) = \mathbf{o} + D\,(\mathbf{v} \odot \mathbf{s}), \qquad \phi^{-1}(\mathbf{x}) = D^{-1}(\mathbf{x} - \mathbf{o}) \oslash \mathbf{s},$$

where $\odot$ and $\oslash$ are elementwise product and quotient. **Both label generation and evaluation use** ϕ **and** ϕ^{-1}, so a training target and its evaluation target are the same physical point.

Intensities are windowed to the pulmonary range and rescaled to 8 bits,

$$\tilde{V}(\mathbf{v}) = \left\lfloor 255 \cdot \frac{\mathrm{clip}(V(\mathbf{v}), h_{\min}, h_{\max}) - h_{\min}}{h_{\max} - h_{\min}} \right\rfloor, \qquad (h_{\min}, h_{\max}) = (-1000, 400)\ \mathrm{HU}.$$

Let $\tilde{V}_z \in \{0, \ldots, 255\}^{N_y \times N_x}$ denote axial slice z. The three input representations compared in this study are the channel operators $\Psi_\bullet\colon\ z \mapsto \{0, \ldots, 255\}^{N_y \times N_x \times 3}$

$$\Psi_{2D}(z) = [\tilde{V}_z,\ \tilde{V}_z,\ \tilde{V}_z], \qquad \Psi_{MIP}(z) = [M_z,\ M_z,\ M_z],\ \ M_z = \max_{|k| \le 1} \tilde{V}_{z+k}, \qquad \Psi_{2.5D}(z) = [\tilde{V}_{z-1},\ \tilde{V}_z,\ \tilde{V}_{z+1}].$$

Ψ_{MIP} is a rank-1 map into channel space: it destroys the through-plane ordering that $\Psi_{2.5D}$ preserves. All three share the same domain, so any performance difference is attributable to the operator.

C. Supervision extent *(Section 2.3)*

Let a reference nodule be $g = (\mathbf{x}_g, d_g)$, a centre in world coordinates and an annotated diameter in millimetres. Model the lesion as the ball $B(\mathbf{x}_g, d_g/2)$. The axial plane at slice index z sits at through-plane offset

$$\delta_z \ = \ \left(z - [\phi^{-1}(\mathbf{x}_g)]_3\right) s_z \quad \text{mm},$$

and intersects the ball in a disc of **chord diameter**

$$d_{\text{eff}}(z) \ = \ 2\sqrt{(d_g/2)^2 - \delta_z^2}, \qquad \text{defined for } |\delta_z| \le d_g/2.$$

The training box on that slice is a square of side

$$\boxed{w_{\text{label}}(z; r, w_{\min}) \ = \ \max\left(r \cdot \frac{d_{\text{eff}}(z)}{s_{xy}},\ w_{\min}\right)}$$

in pixels, where $r \in (0,1]$ is the **supervision-extent ratio** and $w_{\min}$ is the minimum admissible side. $r = 1$ is loose (full-diameter) supervision; $r < 1$ is core-focused (strict) supervision. In the axial plane the supervised area scales as r^2: strict supervision at $r = 0.6$ covers 36% of the loose area.

The clamping regime

Equation (9) is piecewise. The floor binds exactly when

$$r \cdot \frac{d_{\text{eff}}(z)}{s_{xy}} < w_{\min} \quad \Leftrightarrow \quad d_{\text{eff}}(z) \ < \ \frac{w_{\min}\, s_{xy}}{r} \quad =:\ d^*(r, w_{\min}).$$

Two consequences matter for interpreting the ablation.

1. **r and $w_{\min}$ are not independent factors.** The threshold d^* is proportional to $w_{\min}/r$, so lowering r enlarges the subpopulation whose label is set by the floor rather than by the diameter. Two supervision settings yield *identical* labels only where both are clamped, that is for $d_{\text{eff}} < d^*(1, w_{\min}) = w_{\min}\, s_{xy}$;

between that threshold and $d^*(r, w_{\min})$ only the stricter label is clamped, so the nominal ratio r overstates the contrast actually applied.

2. **The clamped fraction is measurable and must be reported.** Writing $\mathcal{B}$ for the set of all training boxes,

$$\rho(r, w_{\min}) \;=\; \frac{|\{\, b \in \mathcal{B} : d_{\text{eff}}(b) < d^*(r, w_{\min}) \,\}|}{|\mathcal{B}|}$$

is the fraction of the supervision signal that is set by the floor rather than by r. Table 5 of the paper reports ρ for every cell of the $(r, w_{\min})$ grid; it rises monotonically from $\rho = 0.186$ at $r = 1.0$ to $\rho = 0.723$ at $r = 0.4$ (with $w_{\min} = 6$), and from $\rho = 0.249$ at $w_{\min} = 4$ to $\rho = 0.671$ at $w_{\min} = 8$ (with $r = 0.6$). Reporting ρ is what makes the sweep interpretable: a change in CPM across r is a change in the *mixture* of diameter-driven and floor-driven boxes, not in r alone.

Because training and inference run at the native 512×512 resolution, s_{xy} in (9)-(10) is the acquisition in-plane spacing and $w_{\min}$ is exact. (Had the slices been resampled to the detector's 640×640 default, a "6-pixel" floor would correspond to $6\, s_{xy} \cdot 512/640$ mm and would depend on the resize factor, so $w_{\min}$ would carry no scan-independent meaning.)

D. From slice detections to 3D candidates *(Section 2.5)*

The detector f_θ maps a channel image to a set of scored axial boxes,

$$f_\theta\big(\Psi_\bullet(z)\big) = \{(\mathbf{b}_i, p_i)\}_i, \qquad \mathbf{b} = (u, v, w, h) \in [0,1]^4,\; p \in [0,1],$$

retained at $p \geq \tau_0$ after slice-level non-maximum suppression. Whole-volume inference evaluates **every** $z \in \{0, \dots, N_z - 1\}$, so the detection set of a scan is $\mathcal{D} = \bigcup_z \{(z, \mathbf{b}, p)\}$.

Detections are aggregated into 3D candidates by a greedy, confidence-ordered grouping. Order $\mathcal{D}$ so that $p_1 \geq p_2 \geq \cdots$; maintain a set of candidates $\mathcal{C}$, each represented by its most confident member $(z_c, \mathbf{b}_c, p_c)$. Detection j joins candidate c when

$$|z_j - z_c| \leq \Delta_z \quad \text{and} \quad \text{IoU}\big(\mathbf{b}_j, \mathbf{b}_c\big) \;\geq\; \tau_{\text{IoU}},$$

otherwise it seeds a new candidate. Because detections arrive in non-increasing confidence, the representative of a candidate never changes after creation, and the candidate score is

$$p_c \;=\; \max_{j \in c} p_j.$$

The candidate centre is mapped back to world coordinates with (5),

$$\mathbf{x}_c \;=\; \phi\Big(\big(u_c N_x - 1/2\,,\; v_c N_y - 1/2\,,\; z_c\big)\Big),$$

the exact inverse of the encoding used to write the training labels, so no half-voxel bias is introduced between supervision and scoring.

Constants: $\tau_0 = 0.01$, $\Delta_z = 3$ slices, $\tau_{\mathrm{IoU}} = 0.1$. They are identical for every configuration, so they cannot favour one.

E. Uncertainty and paired inference *(Section 2.6)*

Let $\mathcal{S}^{(b)}$, $b = 1, \ldots, B$, be scan-level bootstrap resamples drawn with replacement from $\mathcal{S}$, $|\mathcal{S}^{(b)}| = |\mathcal{S}|$, and let $\mathrm{CPM}_m^{(b)}$ be configuration m's CPM recomputed on $\mathcal{S}^{(b)}$. The percentile interval is $\left[Q_{2.5}\{\mathrm{CPM}_m^{(b)}\},\ Q_{97.5}\{\mathrm{CPM}_m^{(b)}\}\right]$.

Crucially, **all configurations are evaluated on the same resample $\mathcal{S}^{(b)}$**. The bootstrap replicates of a difference are therefore paired, and

$$\Delta_{m,m'}^{(b)} \;=\; \mathrm{CPM}_m^{(b)} - \mathrm{CPM}_{m'}^{(b)}$$

has a well-defined percentile interval $\left[Q_{2.5}\{\Delta^{(b)}\},\ Q_{97.5}\{\Delta^{(b)}\}\right]$ and a two-sided bootstrap p-value $2\min\left(\Pr\left[\Delta^{(b)} \le 0\right], \Pr\left[\Delta^{(b)} \ge 0\right]\right)$. This is the quantity round 1 did not report: non-overlap of two marginal intervals is a strictly weaker statement than an interval on the difference.

Partition sensitivity is assessed separately. With $\mathrm{CPM}_m^{(k)}$ the CPM computed inside official fold k's own test subset, the paired fold-wise difference $\delta^{(k)} = \mathrm{CPM}_m^{(k)} - \mathrm{CPM}_{m'}^{(k)}$ is summarised by its mean, its median, the win count $\sum_k \mathbf{1}\left[\delta^{(k)} > 0\right]$, a Wilcoxon signed-rank test and the paired effect size $d_z = \bar{\delta}/\mathrm{sd}(\delta)$. Training stochasticity is assessed by repeating a configuration over seeds $\sigma \in \Sigma$ and reporting $\mathrm{mean}_\sigma \mathrm{CPM}$ and $\mathrm{sd}_\sigma \mathrm{CPM}$. The three sources - evaluation-sample noise (15), partition noise ($\delta^{(k)}$) and optimisation noise (Σ) - answer different questions and are reported separately rather than collapsed into one interval.
